# *Meloidogyne javanica* fatty acid- and retinol-binding protein (Mj-FAR-1) regulates expression of lipid-, cell wall-, stress- and phenylpropanoid-related genes during nematode infection of tomato

**DOI:** 10.1186/s12864-015-1426-3

**Published:** 2015-04-08

**Authors:** Ionit Iberkleid, Noa Sela, Sigal Brown Miyara

**Affiliations:** Department of Entomology, Nematology and Chemistry Units, Agricultural Research Organization (ARO), Volcani Center, P.O. Box 6, Bet Dagan, 50250 Israel; Department of Plant Pathology and Microbiology, Faculty of Agriculture, Food and Environment, Hebrew University of Jerusalem, Rehovot, 76100 Israel

**Keywords:** Fatty acid- and retinol-binding protein, Nematode–host interaction, RNA-Seq, Plant defense mechanism, *Meloidogyne*, Parasitism, Effector

## Abstract

**Background:**

The secreted *Meloidogyne javanica* fatty acid- and retinol-binding (FAR) protein Mj-FAR-1 is involved in nematode development and reproduction in host tomato roots. To gain further insight into the role of Mj-FAR-1 in regulating disease development, local transcriptional changes were monitored in tomato hairy root lines with constitutive *mj-far-1* expression compared with control roots without inoculation, and 2, 5 and 15 days after inoculation (DAI), using mRNA sequencing analysis.

**Results:**

Gene-expression profiling revealed a total of 3970 differentially expressed genes (DEGs) between the two lines. Among the DEGs, 1093, 1039, 1959, and 1328 genes were up- or downregulated 2-fold with false discovery rate < 0.001 in noninoculated roots, and roots 2, 5, and 15 DAI compared with control roots, respectively. Four main groups of genes that might be associated with Mj-FAR-1-mediated susceptibility were identified: 1) genes involved in biotic stress responses such as pathogen-defense mechanisms and hormone metabolism; 2) genes involved in phenylalanine and phenylpropanoid metabolism; 3) genes associated with cell wall synthesis, modification or degradation; and 4) genes associated with lipid metabolism. All of these genes were overrepresented among the DEGs. Studying the distances between the treatments, samples from noninoculated roots and roots at 2 DAI clustered predominantly according to the temporal dynamics related to nematode infection. However, at the later time points (5 and 15 DAI), samples clustered predominantly according to *mj-far-1* overexpression, indicating that at these time points Mj-FAR-1 is more important in defining a common transcriptome.

**Conclusions:**

The presence of four groups of DEGs demonstrates a network of molecular events is mediated by Mj-FAR-1 that leads to highly complex manipulation of plant defense responses against nematode invasion. The results shed light on the *in vivo* role of secreted FAR proteins in parasitism, and add to the mounting evidence that secreted FAR proteins play a major role in nematode parasitism.

**Electronic supplementary material:**

The online version of this article (doi:10.1186/s12864-015-1426-3) contains supplementary material, which is available to authorized users.

## Background

Among the most devastating plant-parasitic nematodes are the sedentary *Meloidogyne* root-knot nematodes (RKNs), which are obligate biotrophs [1]. These parasites interact with their hosts in a subtle and sophisticated manner that is achieved by sustaining a constitutive dialog with select host cells in the vascular cylinder. These cells are the nematode feeding sites, termed giant cells (GCs), upon which nematode development and reproduction rely [2-4]. Although the mechanism by which RKNs establish the GC system is unknown, increasing evidence indicates that glandular secretions (effectors) injected into plant cells by the nematodes interact directly or indirectly with essential plant components, leading to the establishment and maintenance of nematode feeding sites [5-9]. Two esophageal gland types are involved in producing effectors: two subventral glands and one dorsal gland [6]. Other organs in contact with the external environment that produce secretory proteins include the amphids and cuticle. In the last two decades, several cuticle proteins from plant-parasitic nematodes have been identified, including some that are important for parasitism [10-13]. To ensure successful nematode development and reproduction, the nematode-induced feeding-site structure must be maintained for up to 6 weeks, which requires continuous suppression of the plant defense response throughout this period. Suppression of plant defense genes after RKN infection has been demonstrated in microarray studies [14], but the mechanism governing this suppression remains elusive. Two major pathogen-defense-signaling pathways that have been extensively studied are the salicylic acid (SA)-dependent pathway and a SA-independent pathway that involves jasmonic acid (JA) and ethylene (ET) [15,16]. These pathways crosstalk via a complex network of regulatory interactions, and are susceptible to continuous manipulation by plant pathogens for the promotion of virulence and disease production [16].

A group of proteins termed fatty acid- and retinol-binding (FAR) proteins, secreted by all trophic groups of nematodes, have long been acknowledged for their potential function in host immunomodulation [17-23]. FAR proteins are of major interest for several reasons. i) They may play an important role in scavenging fatty acids and retinol for the survival of the parasite [20]. ii) They may induce localized depletion of essential lipids such as oxylipins, thereby compromising the host’s defensive immune response [11,24]. iii) They are located at the host–parasite interface [18]. iv) Their structure is unique to the nematode phylum and is unlike that of any other known family of lipid-binding proteins [18,19,21,25]. Together with their presence in multiple families of parasitic nematodes, these findings lend support to the notion that this nematode-restricted family of proteins plays a crucial role in host parasitism [18].

The role played by plant-parasitic FAR proteins in negating the plant’s defense response was first studied for the potato cyst nematode *Globodera pallida* FAR (Gp-FAR-1) demonstrating lipid-binding activity of Gp-FAR-1 to linoleic and linolenic acids, and inhibition of lipoxygenase (LOX)-mediated modification of these substrates *in vitro* [11]. More recently, a functional analysis of the role of *Meloidogyne javanica* FAR (Mj-FAR-1) in RKN–plant interactions was performed [26]. The spike in expression of *mj-far-1* by the parasitic nematode *M. javanica* second-stage juveniles (J2) at 3–5 days after inoculation (DAI), together with its abundant deposition in the apoplast during the sedentary stages, suggests a primary role for this effector protein in the early and late stages of the host–parasite interaction. Moreover, constitutive expression of *mj-far-1* in tomato (*Solanum lycopersicum*) hairy roots renders plants more susceptible to infection by *M. javanica* [26]. Increased host susceptibility to nematode infection following the overexpression of nematode parasitism genes has been documented [27,28], suggesting that an excess of some effector proteins enhances a compatible host–parasite interaction via modulation of the plant stress [28] and defense [27] responses. Despite extensive research into the functional role of plant-parasitic FAR proteins [11,26], little is known about the molecular mechanisms underlying the increased susceptibility response in *mj-far-1*-expressing roots. To further clarify the increased susceptibility in a root line expressing *mj-far-1* in response to *M. javanica* infection, we analyzed gene expression in roots of transgenic tomato differing in their constitutive expression of the nematode *mj-far-1*. Many of the genes that were differentially regulated in *mj-far-1*-expressing roots were tomato genes known to play important roles in pathogen-mediated defense responses. These responses involve physicochemical processes, such as cell wall regulation and modification, and biochemical responses such as biosynthesis and regulation of compounds associated with fatty acids and the phenylpropanoid-signaling pathways. Our results provide insights into the transcription-regulation events, driven by Mj-FAR-1 secreted by the invading nematode, that facilitate nematode development and disease production in the host plant.

## Results

### Transcriptomic data collection and analysis

**Figure 1:**
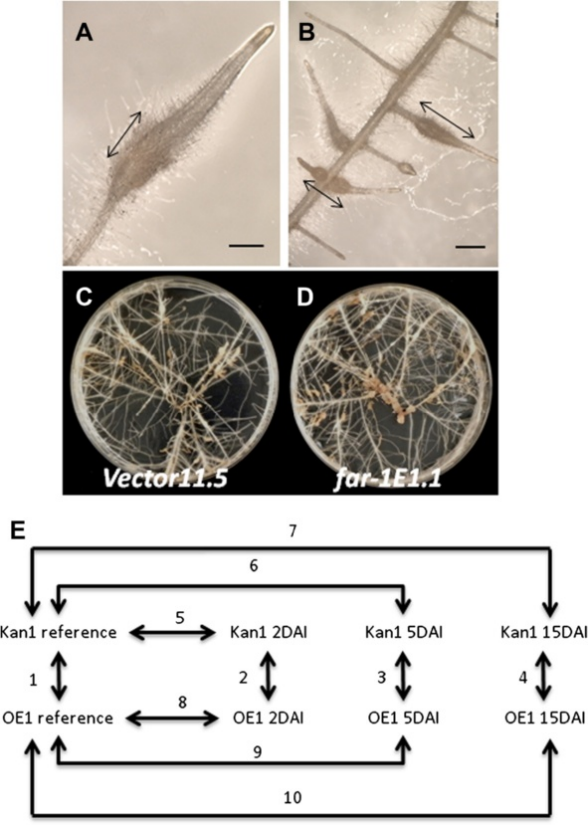
**Plant material used for RNA-Seq analysis and the experimental design for complete tomato RNA-Seq profiling of tomato root lines carrying the kanamycin-resistance gene (Kan) or overexpressing** ***mj-far-1*** **(OE) inoculated with** ***Meloidogyne javanica*** **.** A representative 5 DAI gall used for RNA extraction on roots of the tomato control Kan **(A)** and OE line **(B)** following inoculation with *Meloidogyne javanica*. Arrows indicate segments collected for RNA extraction. Bars = 150 μm. Late infection stage of the tomato control Kan line **(C)** and OE line **(D)** as shown at 15 DAI. Note the increased size and density of galls on the OE vs. Kan roots. **(E)** Schematic representation of the experimental procedure. Four comparisons (1–4) between control Kan and OE lines were performed at each time point: 0, 2, 5, and 15 DAI. In addition, the reference tissue of each root line was directly compared with the transcript profile of the same root line at 2, 5, and 15 DAI: comparisons 5–7 for Kan and 8–10 for OE lines.

Our experimental system exploited the higher susceptibility of roots overexpressing *mj-far-1* upon *M. javanica* infection [26] to characterize *mj-far-1*-mediated differences in gene expression during the *M. javanica* infection process. Root samples of vector 11.5 carrying the kanamycin-resistance gene (Kan control roots) and *mj-far-1.1* lines overexpressing *mj-far-1* (OE roots) from *in vitro*-infected tomato root cultures were harvested at 2, 5, and 15 DAI. Equivalent root segments from noninoculated root cultures of both lines were used as reference root tissues. At 5 DAI, the harvested samples of root tips or segments showed prominent swelling, an indication of nematode invasion and establishment (Figure 1A,B). At 15 DAI, mature galls on primary roots were hand-dissected (Figure 1C,D). As reported previously [26], accelerated disease development was observed for OE roots compared with Kan roots, as indicated by increased gall incidence (Figure 1C,D). To monitor the expression levels of differentially expressed genes (DEGs) with disease progression, and to evaluate the effect of *mj-far-1* overexpression, which underlies the increase in susceptibility, changes in gene expression were investigated by directly comparing noninoculated OE and Kan roots (Figure 1E, comparison 1). Similarly, expression profiles of OE and Kan roots were compared at designated time points after inoculation (Figure 1E, comparisons 2–4). In addition, noninoculated Kan and OE root gene-expression profiles were compared to those upon inoculation of the same root line at 2, 5, and 15 DAI (Figure 1E, comparisons 5–10). RNA was extracted for transcriptome analysis as described in Experimental procedures and RNA-Seq was performed on the Illumina HiSeq™ 2000 platform, yielding an average of 26.6 million high-quality reads per sample (Table 1). Paired-end transcript sequences were mapped against the International Tomato Annotation Group (ITAG) *Solanum lycopersicum* protein reference version 2.3 (http://solgenomics.net) with SOAPaligner/SOAP2 [29]. Gene expression was quantified as the total number of reads (paired-end) from each sample that uniquely align to the transcriptome reference of ITAG2.3 using the aligner SOAP2. An average 20.1 million reads from paired-end sequencing uniquely aligned to the reference sample, and represented an average of 75.6% of the total reads (Table 1) used in the bioinformatics analysis.

**Table 1:** **Alignment of RNA-Seq reads to the ITAG2.3 reference transcriptome**

**Treatment**	**Non-inoculated**		**2 DAI**		**5 DAI**		**15 DAI**	
**Root lines**	**OE**	**KAN**	**OE**	**KAN**	**OE**	**KAN**	**OE**	**KAN**
**High Quality Paired-End Reads**	27,267,694	27,277,056	25,641,052	26,805,144	26,170,776	26,498,432	26,382,764	26,932,422
**Uniquely Aligned Reads**	20,798,101	20,941,168	19,677,396	20,722,903	19,444,292	20,150,007	19,296,181	20,207,686
**Total Unmapped Reads**	6,071,686	5,911,916	5,568,661	5,679,513	6,306,052	5,877,328	6,581,004	6,168,739

The number of paired-end and uniquely aligned sequence reads analyzed from Illumina sequencing runs of all eight samples are arranged by treatment type (noninoculated, and 2, 5, and 15 DAI) and root line.

### *mj-far-1*-mediated differences in gene expression

**Figure 2:**
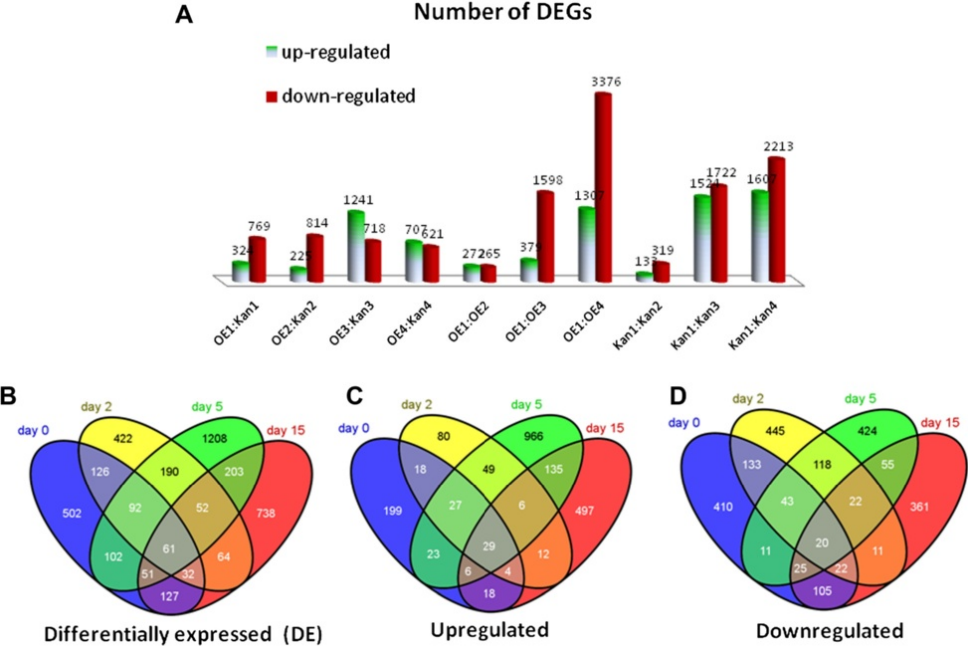
**Regulation of differentially expressed genes among inoculated, noninoculated of** ***mj-far-1*** **overexpressing and control roots. (A)** Comparison of differentially regulated genes from RNA-Seq data between tomato root lines at each designated time point. Numbers of up- and downregulated genes are indicated from 10 comparisons. **(B)** A generalized Venn diagram showing intersection of genes that are differentially regulated (up- and downregulated) in *mj-far1.1* root line compared with vector 11.5 control roots among noninoculated, 2, 5, and 15 DAI samples. **(C)** Intersection of genes that are upregulated in the *mj-far1.1* root line compared with vector 11.5 control roots among noninoculated, 2, 5, and 15 DAI samples. **(D)** Intersection of genes that are downregulated in *mj-far1.1* root line compared with vector 11.5 control roots among noninoculated, 2, 5, and 15 DAI samples. Genes present in two sets are shown in the intersection, so that the sum of the numbers within a circle is the total number of genes in that set. The size of the circles is not representative of the quantity of probe sets. Overlapping areas represent common probe sets. Fold change with an absolute value >2 and *P* value ≤ 0.05 was used for the analyses.

To assess the regulation of tomato transcripts by Mj-FAR-1, differential expression analysis (see Figure 1E for all comparisons conducted) was performed between inoculated and noninoculated OE and Kan root lines. For the calling of differentially regulated genes, the false discovery rate (FDR) threshold was set to ≤0.001 and log2 ratio ≥ 1. The number of DEGs, both upregulated (>1-fold) and downregulated (<1-fold), increased with time after inoculation for both OE and Kan root lines (Figure 2A). Based on Venn diagrams (Figure 2B–D), a total of 3970 DEGs were identified in OE compared with Kan root lines using FDR ≤ 0.001. The number of DEGs common to the OE root line alone and the OE root–nematode interactions is indicated in the overlapping portions of the circles. Of the 3970 genes, 2069 were upregulated and 2205 were downregulated in OE vs. Kan lines for all inoculated and noninoculated samples. The numbers of upregulated genes in noninoculated OE samples and those at 2, 5, and 15 DAI compared with the Kan roots at the same time points were 324, 225, 1241, and 707, respectively (Figure 2C). The numbers of downregulated genes of noninoculated OE samples and those inoculated at 2, 5, and 15 DAI compared with Kan roots were 769, 814, 718, and 621, respectively (Figure 2D). A total of 61 upregulated and downregulated genes overlapped between all noninoculated and inoculated OE root samples (Figure 2B, Table 2). These genes might contribute to the *mj-far-1*-associated increase in susceptibility in the *mj-far1.1* root line. Among this group were genes involved in fatty acid metabolism, such as those encoding the long-chain fatty acid-CoA ligase (Solyc08g008310.2.1) and the lipid-modification enzyme lipase (Solyc05g018770.1.1). In addition, a group of hormone signal-related genes that were differentially regulated in OE roots included JA-related genes, such as a gene encoding a proteinase inhibitor (Solyc03g098710.1.1), and auxin-related genes, such as the gene encoding indole-3-acetic acid-amido synthetase (Solyc02g092820.2.1). Other genes involved in plant defense that encoded the WRKY transcription factor (Solyc05g053380.2.1), a pathogenesis-related (PR) gene (Solyc09g007020.1.1), and a gene involved in the phenylalanine pathway (Solyc02g081800.1.1) showed consistent differential regulation among the treatments (Table 2). An additional group of genes that might shed light on *mj-far-1* regulation of gene expression constituted DEGs unique to inoculated samples, in which a total of 52 upregulated and downregulated genes overlapped only among inoculated OE roots (Table 3). This group included genes involved in cell wall modification and remodeling, such as those encoding expansin-like proteins (Solyc08g07790 0.2.1and Solyc03g093390.2.1) and cell wall protein (CWP) (Solyc09g097770.2), hormone-related genes such as those encoding auxin-responsive protein (Solyc08g021820.2.1) and gibberellin synthesis (Solyc12g042980.1.1), a gene of the phenylpropanoid pathway encoding chalcone synthase (CHS) (Solyc05g053550.2.1), and defense-related genes such as those encoding pathogenesis-related proteins (Solyc07g006710.1.1 and Solyc01g106640.2.1). Changes associated with fatty acid metabolism that were restricted to the inoculated root samples included genes encoding the fatty acid elongase 3-ketoacyl-CoA synthase (Solyc03g005320.2.1) and the nonspecific lipid-transfer protein (Solyc06g054070.2.1) (Table 3).

**Table 2:** **Differentially expressed genes common to noninoculated and inoculated** ***mj-far-1*** **-overexpressing root line and their functional categories**

**Functional categories**	**Sub-category**	**Gene ID**	**OE1-KAN1**	**OE2-KAN2**	**OE3-KAN3**	**OE4-KAN4**	**Annotation**
**Cell Wall**	Proteins LLR	Solyc07g053840.1.1	-2.44	-2.20	-9.83	-2.24	LRP receptor-like serine/threonine-protein kinase , RLP
	Cellulose synthesis COBRA	Solyc03g114900.2.1	-1.22	-1.19	-2.29	-1.46	COBRA-like protein
	Degradation pectate lyase and polygalacturonases	Solyc12g096750.1.1	-2.32	-3.37	-1.90	-7.01	Polygalacturonase 4
	Modification	Solyc10g086520.1.1	-1.23	-1.49	-1.81	-1.02	Expansin-1
	UDP glucosyl and glucoronyl transferase	Solyc05g053400.1.1	2.20	2.57	1.15	3.53	Glucosyltransferase
**Cell organization**	Chloroplast location	Solyc06g062400.1.1	2.12	1.65	2.26	1.70	Cloroplast unusual positioning 1A
**Development**	Nodule formation	Solyc05g055540.1.1	2.94	1.64	2.54	1.99	Nodulin family protein
	unkown	Solyc06g082460.1.1	-1.90	-1.29	-1.11	-1.46	Plant-specific domain TIGRO1568 family protein
**Hormone metabolism**	Auxin induced/regulated/responsive	Solyc02g092820.2.1	-2.99	-1.80	-4.29	-5.59	Indole-3-acetic acid-amido synthetase GH3.8
		Solyc12g005310.1.1	-2.75	-1.18	-3.60	-4.05	Auxin-responsive GH3-like
	Ethylene synthesis/degradation	Solyc09g010000.2.1	1.42	1.80	1.87	1.11	1-aminocyclopropane-1-carboxylate oxidase-like protein
		Solyc11g072310.1.1	1.51	1.45	1.41	1.72	Gibberellin 20 -oxidase-3
		Solyc06g073580.2.1	-3.78	-1.50	-1.01	1.16	1-aminocyclopropane-1-carboxylate oxidase-like protein
	Jasmonic acid induced/regulated/responsive	Solyc03g098710.1.1	-4.38	-1.50	-2.09	2.49	Kunitz-type proteinase inhibitor A4 (Fragment)
		Solyc03g098720.2.1	-10.39	-1.72	-10.48	4.26	Kunitz trypsin inhibitor
**Lipid metabolism**	FA Synthesis and FA Elongation	Solyc08g008310.2.1	-3.21	-2.93	-3.52	-3.27	Long-chain-fatty-acid-CoA ligase
	Phospholipid synthesis	Solyc12g040790.1.1	-1.07	-1.19	-1.40	2.19	Menaquinone biosynthesis methyltransfere ubiE
	Lipid modification	Solyc05g018770.1.1	-2.58	-12.08	-10.93	-4.19	Esterase/lipase/thioesterase (Fragment)
**N-metabolism/degradation**	Glutamate dehydrogenase	Solyc05g052100.2.1	-5.33	-11.68	-2.12	-1.56	Glutamate dehydrogenase
	Phosphotransfer and Pyrophoshatases guanylate kinase	Solyc10g79140.1.1	1.30	1.75	2.57	2.48	Guanylate kinase
	NUDIX hydrolases	Solyc07g045430.2.1	2.34	2.61	1.62	-2.97	Nudix hydrolase 2
**Polyamine metabolism**	SAM/decarboxylase	Solyc06g054460.1.1	-1.37	-2.12	-1.17	-1.96	S-adenosylmethionine decarboxylase proenzyme
	Spermidine/synthase	Solyc06g053510.2.1	-5.21	-2.87	-1.88	1.21	Spermidine synthase
**Protein metabolism**	Protease	Solyc05g016250.2.1	-11.21	-11.18	-11.08	-9.72	Cysteine-type peptidase
		Solyc07g066500.1.1	11.02	9.87	4.88	11.14	U1p1 protease family C-terminal catalytic domain containing protein
		Solyc01g057880.1.1	-4.10	-5.48	-3.35	-4.10	U1p1 protease family C-terminal catalytic domain containing protein
	Postranslational modification	Solyc08g066400.1.1	-10.91	-10.97	-10.39	-10.64	Protein kinase (Fragment)
		Solyc03g083800.1.1	5.01	10.74	5.59	11.45	Serine/threonine-protein phosphatase 7 long form homolog
	Targeting	Solyc12g096550.1.1	1.20	1.10	1.23	1.11	Pheophorbide a oxygenase family
**Transcription factor**	GeBP like	Solyc07g052900.1.1	1.21	1.55	1.66	1.96	Os09g0451700 protein (Fragment)
		Solyc07g052700.2.1	2.84	2.57	4.21	2.30	MADS-box transcription factor 1
	MADS box transcription factor/family	Solyc02g089200.2.1	4.79	3.11	5.74	4.14	MADS-box transcription factor
		Solyc07g052720.2.1	3.67	2.31	4.65	2.99	MADS-box protein AGL66
	Putative transcription regulator	Solyc10g051140.1.1	-1.94	-2.58	-1.90	-1.81	Genomic DNA chromosome 5 P1 clone MTE17
	WRKY domain transcription factor family	Solyc05g053380.2.1	-2.30	-1.99	1.60	-2.02	WRKY transcription factor 31
	unknown	Solyc01g100440.1.1	2.07	1.33	1.89	2.42	Transcription regulatory protein SNF5
**Secondary metabolism**	Isoflavone reductase	Solyc10g052500.1.1	-4.36	-510	-3.32	-4.16	Phenylcoumaran benzylic ether reductase 3
	Isoprenoids/terpenoids	Solyc10g005390.2.1	1.86	1.47	2.96	2.67	Linalool synthase
	Phenylpropanoids	Solyc02g08100.1.1	2.24	2.86	3.13	1.41	Acyltransferase (Fragment)
**Signaling**	Sugar and nutrien phisiology	Solyc12g099780.1.1	-1.69	-1.48	1.91	1.05	Unknown Protein
	Receptor kinase leucine rich repeat XI	Solyc12g009780.1.1	8.74	3.37	3.18	8.43	LRR receptor-like serine/ threonine-protein kinase, RLP
**Abiotic stress**	Heat stress	Solyc06g011400.2.1	4.54	2.57	4.40	2.45	ATP-dependent chaperone c1pB
		Solyc06g11380.2.1	5.99	3.67	4.70	2.51	Chaperone C1pB
		Solyc06g011370.2.1	3.86	3.46	3.52	2.25	Chaperone protein clpB 2
**Plant defence**	Pathogen attack response	Solyc09g007020.1.1	1.70	1.24	1.07	1.14	Pathogenesis-related protein
	Pathogen resistance	Solyc07g009510.1.1	2.56	2.44	1.76	1.05	Chitinase
	Gene to gene resistance/recognation	Solyc12g044190.1.1	1.07	1.33	2.37	2.18	Nbs-lrr, resistance protein
	Redox state	Solyc01g081250.2.1	1.22	1.02	1.34	1.08	Glutathione-S-transferase
		Solyc03g116120.1.1	2.80	1.87	2.18	1.99	Glutathione S-transferase 12
		Solyc08g014330.2.1	-2.24	-1.19	-1.06	1.08	Primary amine oxidase
	unknown	Solyc01g017600.2.1	-2.17	-5.86	-10.57	1.48	Plant viral-response family protein
**Transport**	Amino acids	Solyc01g100390.2.1	2.12	2.04	2.63	1.21	Pyrophosphate-energized proton pump
	Peptides and oligopeptides	Solyc03g113430.2.1	1.63	1.24	3.70	1.28	Peptide transporter
	unknown	Solyc01g73670.2.1	-1,60	-1.63	-1.98	1.22	Uncharacterized MFS-type transporter C19orf28
	Protease inhibitor/seed storage /lipid transfer protein (LTP) family protein	Solyc03g083990.1.1	-2.08	1.15	1.25	-2.66	Cortical cell-delineating protein
**Not assigned**	unknown	Solyc04g015610.2.1	-1.89	-1.27	-1.50	-3.73	Os01g0611000 protein (Fragment)
		Solyc10g080380.1.1	-1.53	-3.73	-3.61	-1.55	Unknown Protein
		Solyc07g007770.1.1	-6.36	-5.19	-5.23	-4.54	Unknown Protein
		Solyc09g091810.1.1	-2.80	-1.29	-1.62	-2.78	Unknown Protein
		Solyc01g056370.2.1	12.79	12.99	13.67	13.64	Unknown Protein
		Solyc05g052880.2.1	1.93	1.37	1.89	3.33	Unknown Protein

Gene ID number and log2 values at each time point before and after inoculation are indicated. All genes were considered to be differentially expressed with a threshold *q*-value < 0.05.

**Table 3:** **Differentially expressed genes upon** ***M. javanica*** **inoculation**

**Functional categories**	**Sub-category**	**Gene ID**	**OE2-KAN2**	**OE3-KAN3**	**OE4-KAN4**	**Annotation**
**TCA/ org. transformation**	Carbonic anhydrases	solyc02g067750.2.1	1.99565	2.704447	2.324205	Carbonic anhydrase
**Cell wall**	Degradation pectate lyases and polygalacturonases	solyc08g068150.2.1	-1.17686	1.198919	1.5876	BURP domain-containing protein
	Modification	solyc08g077900.2.1	-1.5738	-1.34033	-1.07768	Expansin-like protein
		solyc03g093390.2.1	-1.23629	-1.69975	1.122155	Expansin protein
**Lipid metabolism**	FA synthesis and FA elongation	solyc03g005320.2.1	-1.21734	1.118395	1.412513	Fatty acid elongase 3-ketoacyl-CoA synthase
	Lipid degradation	solyc01g100020.2.1	-1.6392	-1.60844	-1.23022	Phospholipase D
**Amino acid metabolism**	Synthesis	solyc01g006620.2.1	-1.39285	1.576919	1.629744	Phosphoribosylanthranilate trnasferase
**Secondary metabolism**	Flavonoids	solyc05g053550.2.1	2.880173	1.665111	-1.64308	Chalcone synthase
	Simple phenols	solyc06go76760.1.1	-1.14152	1.327023	1.041531	Laccase 1a
**Hormone metabolism**	Gibberelin synthesis/degradation	solyc12g042980.1.1	-2.48967	-2.80308	-1.41139	2-oxoglutarate- dependent dioxygenase
**Tetrapyrrole synthesis**	Unspecified	solyc12g005300.1.1	-1.37617	-1.44432	1.099574	Chlorophyllase 2
**Plant Defense**	Gen to resistance/recognition	solyc11g0066401.1	-2.44176	1.276086-	1.705295	Cc-nbs-lrr, resistance protein
		solyc07g006710.1.1	-1.12202	-2.25906	-1.15715	Pathogenesis-related protein PR-1
	Pathogen attack response	solyc01g106640.2.1	-1.43339	-2.26051	-3.62562	Pathogenesis-related protein 1
	Redox state	solyc07g039410.2.1	-2.20729	-1.85636	-1.61107	Nbs-lrr, resistance protein
		solyc05g046030.2.1	-1.44323	-1.95476	-1.75115	Peroxidase
		solyc01g006290.2.1	-4.44176	-2.66733	1.965054	Peroxidase
		solyc01g006310.2.1	-2.19935	-1.68906	-1.71087	Peroxidase
		solyc05g006740.2.1	-1.39462	-1.67555	-1.25058	Glutathione S-transferase
**Miscelaneous**	CytochromeP450	solyc07g052370.2.1	1.592891	1.144126	2.253552	Cytochrome P450
**Transcription factor**	C2c2(Zn) Co-like, Constans-like zinc finger family	solyc07g066510.2.1	-3.16422	-2.54704	2.541796	Zinc finger protein CONSTANS-LIKE 2
	MYB domain transcription factor family	solyc06g005310.2.1	-1.3004	1.639491	1.189045	MYB transcription factor
		solyc10g008700.1.1	-1.65232	2.339096	1.861414	MYB transcription factor
	bZIP transcription factor family	solyc02g072570.1.1	-1.32419	-1.23206	-2.44191	Transcription factor bZIP98
	Aux/IAA family	solyc08g021820.2.1	-10.0373	-2.64797	-4.15734	Auxin responsive protein
	Putative transcription regulator	solyc01g081320.2.1	-1.85679	1.327023	1.545638	Pentatricopeptide repeat-containing protein
**Protein metabolism**	Targeting	solyc07g017520.2.1	-1.36494	1.651521	1.06459	Conserved oligomeric Golgi complex subunit 3
	Posttranslational regulator	solyc04g15120.2.1	-1.7365	-1.3454	-1.44333	U-box domain containing protein expressed
		solyc09g083410.2.1	1.002774	1.290076	1.408902	Amidase hydantoinase/carbamoylase family protein expressed
	Degradation	solyc07g054370.2.1	-1.74661	2.633149	1.463537	F-box/LRR-repeat protein At3g59200
**Signalling**	Receptor kinases	solyc12g005620.1.1	1.813059	1.988114	1.028337	LRR receptor-like serine/threonine-protein kinase,RLP
		solyc06g069740.1.	1.354104	1.251148	-2.06553	Calmodulin-like protein
	Calcium	solyc03g083320.2.1	-1.11983	-1.18853	1.489756	Calcineurin B-like calcium binding protein
		solyc01g097420.1.1	1.390368	1.531115	-1.73009	Calcuim ATPase
	G-proteins	solyc03g078570.2.1	-1.35177	1.730895	1.078512	Ras-related protein Rab-6A
**Transport**	Protease inhibitor/seed storage/lipid transfer protein (LTP) family protein	solyc06g054070.2.1	-1.12655	-1.38876	-1.27683	Non-specific lipid-transfer protein
	Sugars	solyc03g113210.2.1	-2.97781	-10.8452	1.643367	Porin/voltage-dependent anion-selective channel protein
**Not assigned**	Unknown	solyco1g104720.2.1	-3.27322	-3.93722	-2.36617	Unknown Protein
		solyc07g009020.1.1	-2.17045	-4.1934	-1.14893	Unknown Protein
		solyc08g078920.1.1	-1.1221	-1.43749	-1.25381	Proline-rich Protein
		solyc12g049140.1.1	-2.16278	-3.50997	-2.2489	Extensin-like protein Ext1
		solyc06g051500.2.1	-1.31051	3.08695	2.375988	Unknown Protein
		solyc07g008980.2.1	-3.19664	-3.89496	-1.52861	Unknown Protein
		solyc07g009030.2.1	-2.28689	-3.72919	-1.38264	Unknown Protein
		solyc07g032170.2.1	-1.37989	2.61237	1.682212	Abhydrolase domain- containing protein 5
		solyc04g015700.1.1	-1.09406	-2.77071	-1.14691	Unknown Protein
		solyc05g009580.2.1	-1.41924	-1.0847	-1.80119	Aluminum-activated malate transporter-like
		solyc12g014120.1.1	-1.51214	1.122747	1.442261	Unknown Protein
		solyc09g097770.2.1	1.242743	1.587252	1.011286	Cell wall protein
		solyc03g078580.2.1	-1.2353	1.865868	1.701912	Unknown Protein
		solyc06g005210.1.1	2.124099	1.102057	1.169745	Cytochrome P450 like_TBP
		solyc01g097690.2.1	-1.95437	-3.72554	-1.10791	Extensin-like protien Dif54

Gene ID numbers along with log2 values at each time point before and after inoculation are indicated. All genes were considered to be differentially expressed with a threshold *q*-value < 0.05.

Gene ID number along with log2 values at each time point before and after inoculation are indicated. All genes were considered DEGs with a cutoff q-value < 0.05.

### Principal component analysis and distribution of differentially expressed genes

**Figure 3:**
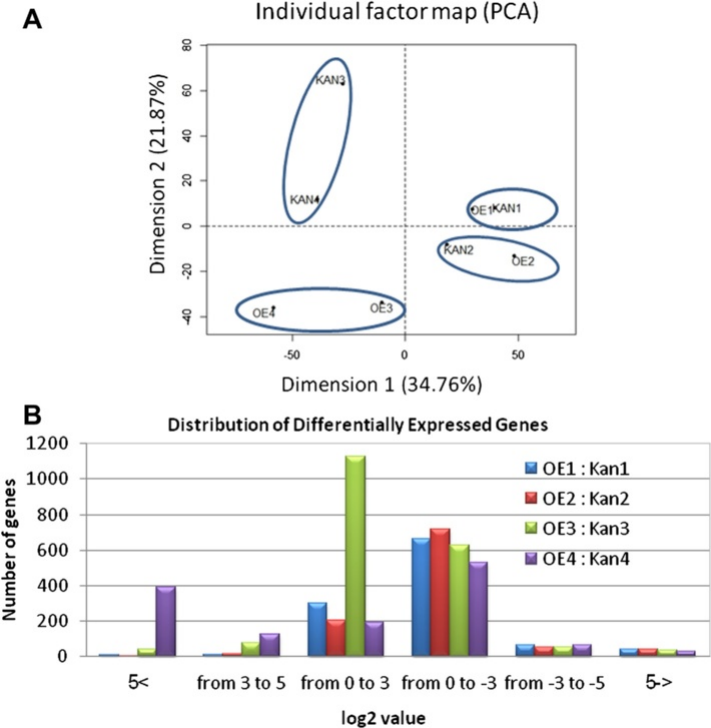
**Distribution of differentially expressed genes. (A)** Three-dimensional representation according to principle component analysis (PCA) of the differential gene expression data of eight treatments used in the RNA-Seq analysis (as implemented in JMP Genomics 5.1). Kan roots are root samples of vector 11.5 carrying the kanamycin-resistance gene (Kan control roots) and OE roots are *mj-far-1.1* lines overexpressing *mj-far-1* (OE roots). In this analysis, samples with similar expression profiles lie closer to each other than those with dissimilar profiles. Axes 1 and 2 show robust class separation into four major groups: Kan1 and OE1; Kan2 and OE2; Kan3 and Kan4; and OE3 and OE4. At the early time points (noninoculated and 2 DAI) the infection itself is responsible for most of the transcriptional variance. However, at 5 and 15 DAI, *mj-far-1* is the variable responsible for most of the transcriptional variance among treatments with infection playing a lesser role. **(B)** Distribution of up- and downregulated differentially expressed genes and their fold change over all comparisons made between OE and Kan root lines.

The Pearson correlation coefficient was used to determine the significance of the correlation between mRNA datasets of all eight samples using R (version 3.0.0) (http://www.R-project.org) in the FactoMineR package [30]. As shown by the principal component analysis (PCA), root profiles of noninoculated and 2 DAI samples clustered with respect to temporal dynamics associated with nematode infection, whereas the effect of *mj-far-1* overexpression was less important at these stages. The resulting dendrogram revealed small differences in the expression levels of DEGs between noninoculated OE and Kan roots as well as between 2 DAI OE and Kan roots (Figure 3A). At 5 and 15 DAI, i.e., once nematode infection had progressed, root samples clustered predominantly in accordance with *mj-far-1* overexpression, revealing broad, global differences in the expression levels of DEGs between OE and Kan root lines (Figure 3A). These results indicate that *mj-far-1* is more important in defining a common transcriptome at later time points (Figure 3A). In this analysis, the most variability in the data was accounted for by dimension 1 (34.76%), while dimension 2 accounted for 21.86% of the variability in the data. Analyzing the distribution of DEGs and measuring the transcriptional changes detected in the OE vs. Kan root lines demonstrated that most of the changes between the lines occurred at 5 DAI when 1959 genes were differentially expressed (Figure 3B).

### Functional categorization of differentially expressed genes

**Figure 4:**
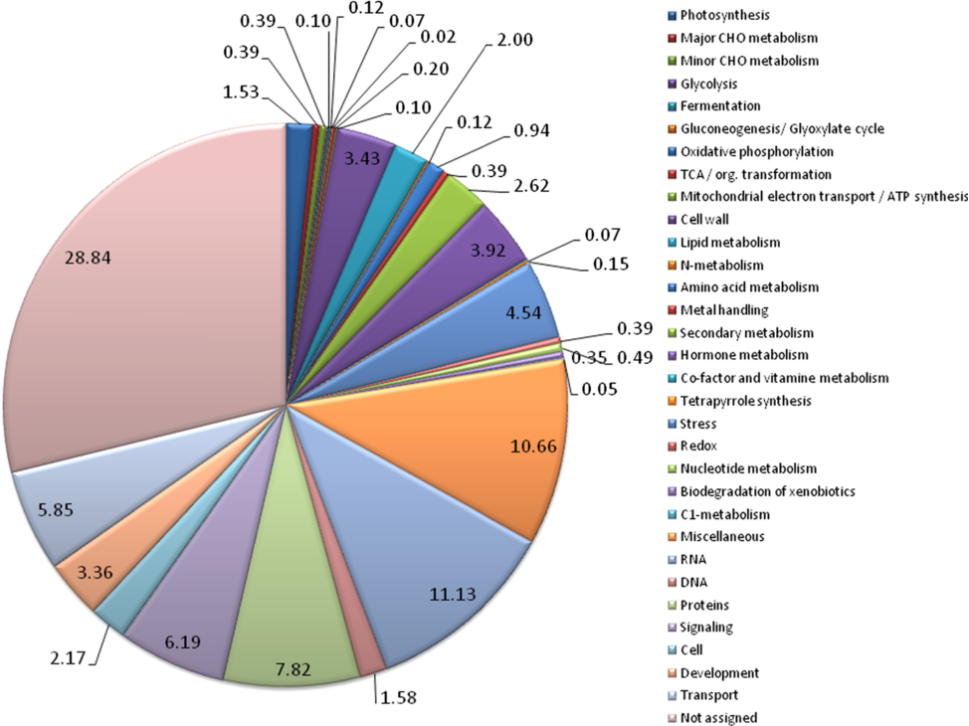
**Functional classification of differentially expressed genes in OE root line vs. Kan root line (** ***q*** **-value < 0.05) among all treatments as illustrated by MapMan categories.**

To obtain an overview of the processes that are altered during the early and late stages of the plant’s response to nematode infection as a consequence of *mj-far-1* overexpression, DEGs were classified using MapMan 2.0.0 [31] (Figure 4). Of the 3970 probe sets, 1144 corresponded to unassigned proteins, i.e., those with no known homolog in *Arabidopsis*. All other probe sets were grouped into functional categories, among them transcripts associated with secondary metabolism (104 probe sets), lipid metabolism (79 probe sets), cell wall (136 probe sets), transport (232 probe sets), hormone (155 probe sets), stress (180 probe sets), development (133 probe sets), and signaling (245 probe sets) (Figure 4). Probe sets that did not fit into any of these categories or fell into multiple categories were grouped as ‘miscellaneous’ (420 probe sets, Figure 4). In this study, we specifically focused on groups that were associated with stress and defense, fatty acids, phenylpropanoids (secondary metabolism), and cell walls. These groups of DEGs were further studied in relation to the increased susceptibility observed in roots overexpressing *mj-far-1*.

### Hormone metabolism- and fatty acid metabolism-related transcripts associated with *mj-far-1* overexpression

Analysis of the ‘hormone metabolism’ category across all time points revealed differential expression of genes related to ET, auxin, methyl jasmonate and SA pathways in OE compared with Kan roots (as shown for 2 DAI in Additional file file 1: Table A1). A detailed analysis demonstrated a remarkable decrease in the expression of ET-related transcripts at 2 DAI in OE compared with Kan roots (Additional file 1: Table A1). These included transcripts encoding 2-oxoglutarate-dependent dioxygenase (Solyc09g089710.2.1), gibberellin 2-beta-dioxygenase (Solyc02g080120.1.1), and gibberellin 20-oxidase 4 (solyc01g093980.2.1), as well as aminocyclopropane-1-carboxylic acid synthase; expression of these transcripts decreased by 1072.16-, 13.03-, 693.7- and 1072.17-fold in OE vs. Kan roots at 2 DAI. Similarly, downregulation of auxin-related genes, such as the gene encoding the PIN6 auxin:hydrogen symporter/transporter (Solyc06g059730.1.1) by 2.83-fold and auxin-responsive family protein (Solyc12g017880.1.1) by 5.26-fold, was observed, together with additional auxin-related transcripts that demonstrated strong downregulation in expression at 2 DAI (Additional file 1: Table A1). With regard to SA-related transcripts, the 6.34-fold increase in expression of salicylic acid carboxyl methyltransferase (Solyc09g091550.2.1) in OE compared with Kan roots at 2 DAI might contribute to the observed root susceptibility [32] (Additional file 1: Table A1).

Given that FAR is implicated in fatty acid metabolism, we next focused on fatty acid-related signaling in OE vs. Kan roots before and throughout the time course of inoculation. Transcript analysis by MapMan showed that the gene encoding the repressor jasmonate ZIM-domain protein 1 (JAZ1) (Solyc12g009220.1.1) was downregulated in noninoculated OE roots. JAZ1 is a nucleus-localized protein belonging to the larger family of TIFY proteins [32] that act as repressors of JA signaling [33,34]. (Similarly, upregulation of the gene encoding allene oxide synthase (AOS) (Solyc01g109150.2.1) was observed in noninoculated OE roots; this protein might induce the JA pathway before inoculation, providing an advantage for RKN infection. At 2 DAI downregulation of a 9-LOX member, the LOXB transcript (Solyc01g099180.2.1; similar to *Arabidopsis* LOX1), by 360-fold and AOS (Solyc04g079730.1.1; similar to *Arabidopsis* AOS) by 4.43-fold was observed, indicating that changes in lipid metabolism are an early response to nematode inoculation. At 5 DAI additional downregulation of 9-LOX (Solyc09g075870.1.1; similar to *Arabidopsis* LOX5) in OE roots compared with Kan roots was observed; although these mentioned isoforms are not known to be involved in JA biosynthesis, their product might be active in local and systemic defense mechanisms against pathogens [35,36] (Table 4). At 15 DAI significant upregulation of 9-LOX (Solyc08g014000.2.1; highly similar to *Arabidopsis* LOX1) and AOS (Solyc10g0079601.1) transcripts was observed in inoculated OE roots compared with the noninoculated control (Table 4). Differential expression of JA- and fatty acid-related transcripts solely as a consequence of nematode infection was studied by analyzing the Kan control roots. Inoculated Kan roots at 5 and 15 DAI showed downregulation of the 9-LOX gene family as LOXB (Solyc01g099200.2.1 and Solyc01g099180.2.1; highly similar to *Arabidopsis* LOX1), and of the 13-LOX family (Solyc05g014790.2.1; similar to *Arabidopsis* LOX6) and as LOXD (Solyc03g122340.2.1; highly similar to *Arabidopsis* LOX3) (Table 4). Similarly, the genes encoding AOS (Solyc11g069800.1.1 and Solyc04g079730.1.1) and 12-oxophytodienoate reductase 3 (Solyc07g007870.2.1) were downregulated. Upregulation of genes encoding hydroperoxide lyase (HPL) (Solyc07g049690.2.1; similar to *Arabidopsis* HPL1), 9-LOX transcripts similar to *Arabidopsis* LOX5 (Solyc09g075870.1.1 and Solyc09g075860.2.1), and LOX1 (Solyc08g014000.2.1 and Solyc01g099210.2.1), AOS (Solyc01g109150.2.1) and OPR2 (12-oxophytodienoate reductase 2) (Solyc01g103390.2.1) was observed. Similar to noninoculated OE samples, in Kan control roots at 5 and 15 DAI transcripts similar to those of the negative regulators of JA signaling JAZ1 (Solyc12g009220.1.1, Solyc07g042170.2.1, Solyc12g049400.1.1) and JAZ2 (Solyc03g122190.2.1) were downregulated as infection proceeded.

**Table 4:** **Dynamics of the gene-expression profile related to the fatty acid pathway**

**Number**	**Function (ITAG)**	**Tomato (NCBI)**	**Arabidopsis thaliana (TAIR)**	**KAN1/ KAN3**	**KAN1/ KAN4**	**OE1/ KAN1**	**OE2/ KAN2**	**OE3/ KAN3**	**OE4/ KAN4**
**solyc05g014790.2.1**	Lipoxygenase	XM_004239145.1 lipoxygenase 6	AT4G15440 - HPL1 (HYDROPEROXIDE LYASE 1)	1.20	1.03	-	-	-	-
**solyc09g075870.1.1**	Lipoxygenase	XM_004247319.1 lipoxygenase 5	AT3G22400 - LOX5 (LIPOXYGENASE 5)	-1.82	-2.15	-	-	-2.06	-
**solyc08g014000.2.1**	Lipoxygenase	NM_001247927 lipoxygenase (LOX1.1)	AT1G55020 - LOX1 (LIPOXYGENASE 1)	-1.32	-	-	-	-	5.48
**solyc01g099210.2.1**	Lipoxygenase	XM_004230159.1 lipoxygenase 5	AT1G55020 - LOX1 (LIPOXYGENASE 1)	-1.75	-2.71	-	-	-	-
**solyc01g099200.2.1**	Lipoxygenase	XM_004231226 lipoxygenase 5	AT1G55020 - LOX1	-	2.34	-	-	-	-
**solyc01g099180.2.1**	Lipoxygenase	XM_004230158 lipoxygenase B	AT1G55020 - LOX1	-	4.20	-	-360.05	-	-
**solyc09g075860.2.1**	Lipoxygenase	XR_183132 lipoxygenase 5	AT3G22400 - LOX5	-	-1.66	-	-	-	-
**solyc03g122340.2.1**	Lipoxygenase	XM_004235501 lipoxygenase (loxD)	AT1G17420 - LOX3 (LIPOXYGENASE 3)	-	1.37	-	-	-	-
**solyc07g049690.2.1**	Cytochrome P450	NM_001247491.1 fatty acid hydroperoxide lyase (HPL)	AT4G15440 - HPL1	-1.18	-	-	-	-	-
**solyc01g109150.2.1**	Cytochrome P450	NM_001247573.1 cytochrome P450 CYP74C4	AT5G42650 - AOS (ALLENE OXIDE SYNTHASE)	-1.79	-3.06	2.98	-	-	-
**solyc11g069800.1.1**	Cytochrome P450	NM_001247904.1 allene oxide synthase (AOS)	AT5G42650 - AOS	-	1.02	-	-	-	-
**solyc04g079730.1.1**	Cytochrome P450	DQ174273.1 allene oxide syntase	AT5G42650 - AOS	-	1.40	-	-4.43	2.74	-
**solyc10g007960.1.1**	Allene oxide synthase	DQ174273.1 allene oxide syntase AJ278331	AT5G42650 - AOS	-	-	-	-	-	7.29
**solyc01g103390.2.1**	Flavin oxidoreductase/NADH oxidase	12-oxophytodienoate reductase 2 (OPR2)	AT1G76690 - OPR2 (12-OXOPHYTODIENOATE REDUCTASE 2)	-1.04	-1.72	-	-	-	-
**solyc11g032130.1.1**	NADPH dehydrogenase 3	XM_004250605.1 12-oxophytodienoate reductase 1-like	AT1G76690 - OPR2	-	-	-	-	2.59	-
**solyc07g007870.2.1**	NADH flavin oxidoreductase/12-oxophytodienoate reductase	NM_001246944 12-oxophytodienoate reductase 3 (opr3)	AT2G06050 - OPR3 (OPDA-REDUCTASE 3)	-	1.06	-	-	-	-
**solyc03g122190.2.1**	Jasmonate ZIM domain 2	NM_001247294.1 salt responsive protein 1 (SRG1)	AT1G74950 - JAZ2, TIFY10B (JASMONATE-ZIM-DOMAIN 2)	1.23	2.07	-	-	-	-
**solyc12g009220.1.1**	Jasmonate ZIM-domain protein 1	NM_001247954 jasmonate ZIM-domain protein 1	AT1G19180 - JAZ1 (JASMONATE-ZIM-DOMAIN PROTEIN 1)	1.97	1.92	-2.39	-	2.42	-
**solyc12g049400.1.1**	Protein TIFY 3B	XM_004252359 TIFY 10A-like	AT1G19180 - JAZ1	2.63	2.66	-	-	-	-
**solyc07g042170.2.1**	Jasmonate ZIM-domain protein 3	XM_004243648.1 TIFY 10A-like	AT1G19180 - JAZ1	-	1.33	-	-	-	-

**Figure 5:**
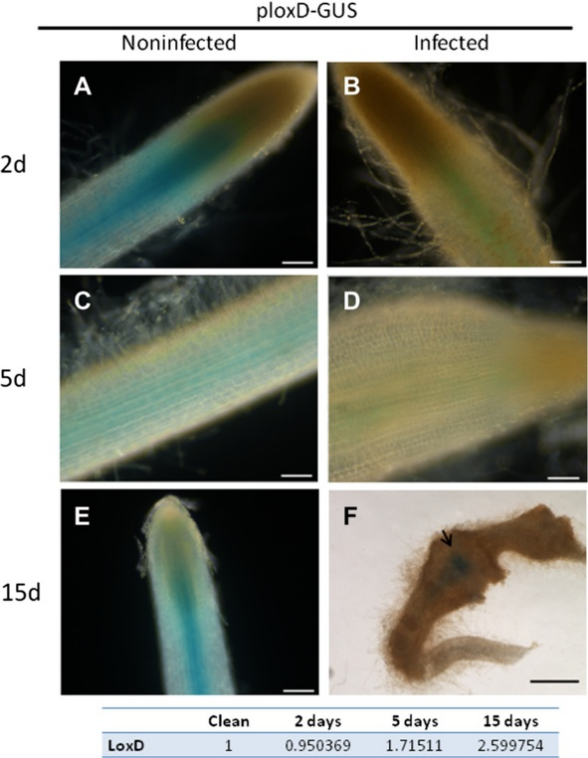
**LOXD promoter (pLOXD)–GUS expression in transgenic tomato hairy root line infected with** ***M. javanica*** **second-stage juveniles (J2s).** Noninfected control roots harboring the pLOXD–GUS fusion construct **(A, C, E)** show GUS staining of the root tip and vascular cylinder. Infected roots harboring pLOXD–GUS **(B, D)** show a decrease in GUS signal at 2 and 5 DAI. However, at 15 DAI **(E)**, GUS signal is observed in the center of the developing gall in the giant cell area induced by the invading nematodes. **(A–E)** Light micrographs as viewed under a light microscope. **(F)** Bright-field image of galls photographed using a stereomicroscope. Bars: **A–E** = 100 μm, **F** = 1000 μm. Differential expression of LOXD in OE roots compared with Kan roots obtained in the RNA-Seq data is shown at the bottom of the table.

Given that the LOXD isoform is involved in JA biosynthesis, we next studied the dynamic expression of LOXD by means of a LOXD promoter–GUS construct. For these experiments, primers corresponding to the 5′ upstream sequences of LOXD (Solyc03g122340.2.1; similar to *Arabidopsis* LOX3) were designed with reference to the recently released genome (ITAG Release 2 [2010-11-28] official annotations on the SL2.31 genome built by ITAG). Promoter fragments were amplified by polymerase chain reaction (PCR) using tomato line 870 genomic DNA as a template, cloned upstream of the GUS reporter gene in the vector pUC19_Y [37], and subsequently cloned in the binary vector pCAMBIA2300 [38]. Transgenic hairy roots were generated in the background of tomato line 870 using the LOXD promoter–GUS construct transformed into *Agrobacterium rhizogenes*. Five positive transgenic hairy roots events were then infected with the avirulent *M. javanica* population. For the LOXD promoter–GUS line (pLOXD–GUS), *LOXD* expression was conspicuous, but restricted to the vascular cylinder, in noninoculated roots (Figure 5A,C,E). Following infection, weak signal corresponding to *LOXD* expression was observed within the vascular tissue at 2 and 5 DAI (Figure 5B,D), which corresponded well with the transcriptome results (Table 4). At 15 DAI, by which time galls had developed, extremely intense signal was detected within the gall, restricted to the vascular tissue associated with GCs (Figure 5F). A similar phenotype was observed for all pLOXD–GUS transformed root events.

### Differential expression of cell wall biosynthesis-, modification-, and remodeling-related genes associated with *mj-far-1* overexpression

**Figure 6:**
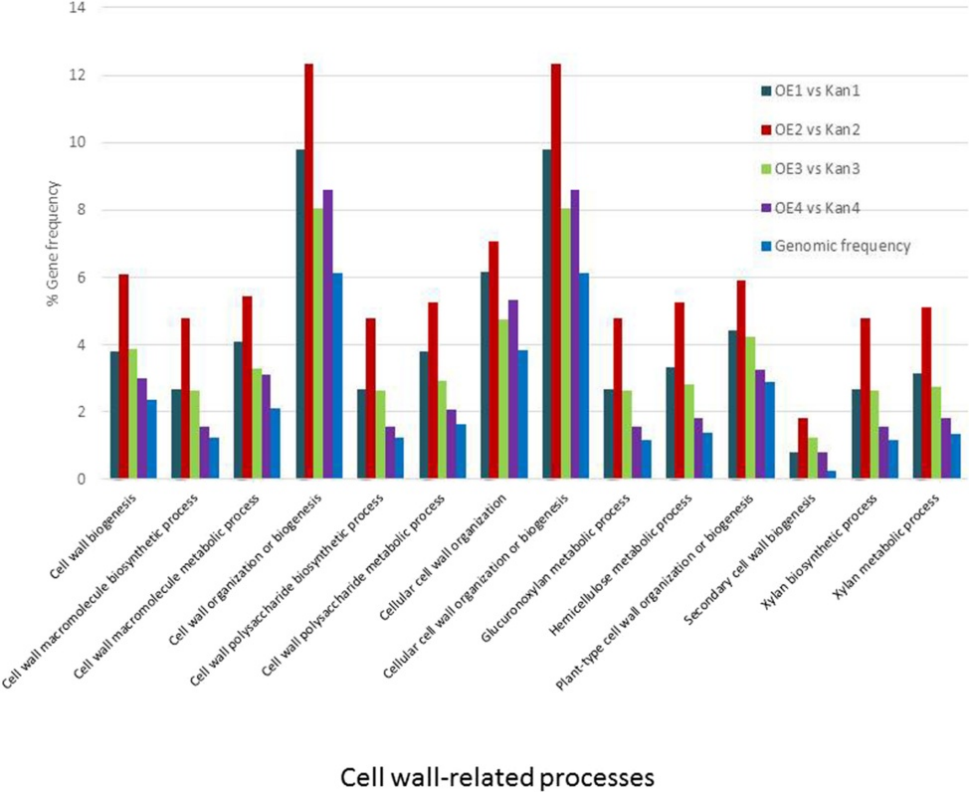
**Frequency distribution of reads putatively associated with cell wall processes identified among differentially expressed genes (DEGs) from the root transcriptomes of all noninfected and infected samples.** All Kan roots are root samples of vector 11.5 carrying the kanamycin-resistance gene (Kan1, Kan2, Kan3, and Kan4 refer to Kan control roots that were noninoculated and at 2, 5, and 15 DAI, respectively) and OE roots are *mj-far-1.1* lines overexpressing *mj-far-1* (OE1, OE2, OE3, and OE4 refer to OE roots that were noninoculated and at 2, 5, and 15 DAI, respectively). Each category of cell wall-related genes is indicated on the *x*-axis and the percentage of genes in each category relative to the ITAG2.3 reference tomato genome and the DEGs is indicated on the *y*-axis.

**Figure 7:**
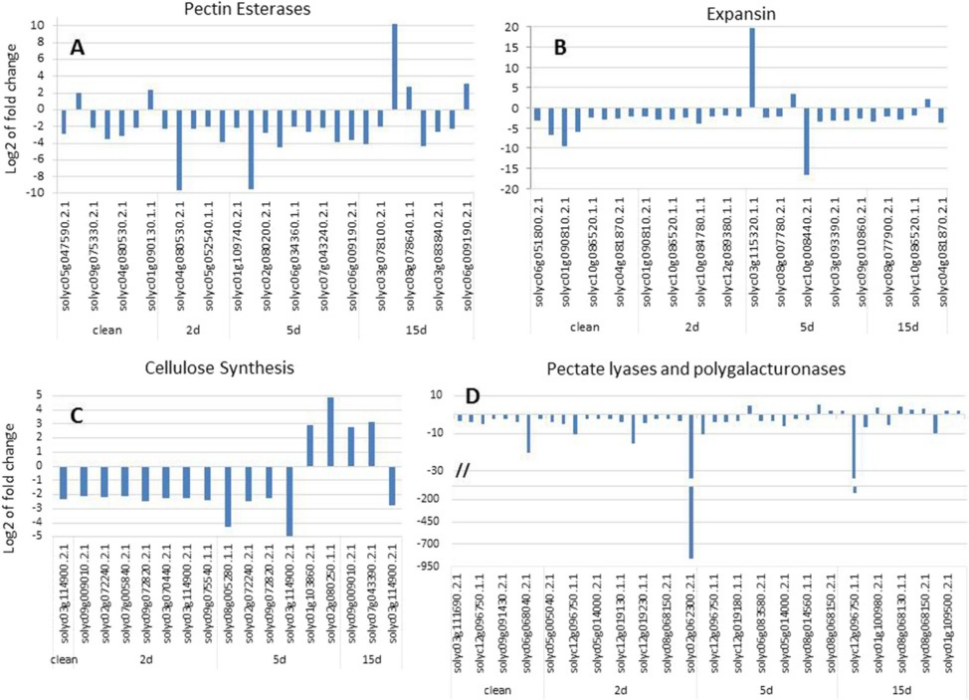
**Regulation of cell wall protein related transcripts.** Fold change in expression of **(A)** pectin esterase-related genes, **(B)** expansin-related genes, **(C)** cellulose synthesis-related genes, **(D)** pectate lyase and polygalacturonase-related genes in OE vs. Kan root lines that were noninoculated and at 2, 5, and 15 DAI.

A distinct difference in the expression of genes showing strong or moderate association with cell wall-related activities was detected in OE vs. Kan roots. This pattern was demonstrated by the high representation of genes belonging to the different cell wall subcategories, as illustrated in Figure 6, whereby transcripts belonging to certain subcategories are overrepresented among the DEGs at a specific time point compared with their frequency in the tomato genome. Among these subcategories, a high representation of cell wall modification- and remodeling-related genes (e.g., those encoding pectin esterases) and expansin-encoding transcripts was observed (Figure 7, Additional file 1: Table A1 and Additional file 2: Table A2). The group of genes associated with cell wall-synthesis activity, such as the gene encoding cellulose synthase (Figure 7, Additional file 1: Table A1 and Additional file 2: Table A2), and cell wall degradation-related genes, such as genes encoding pectate lyases and polygalacturonases, were also overrepresented (Figure 7, Additional file 1: Table A1 and Additional file 2: Table A2). As noted already, at the early time points most genes belonging to these subcategories showed downregulation of the corresponding transcripts in OE roots compared with Kan roots. However, several transcripts belonging to the different subcategories showed remarkable upregulation, in particular at later time points (Figure 7).

**Figure 8:**
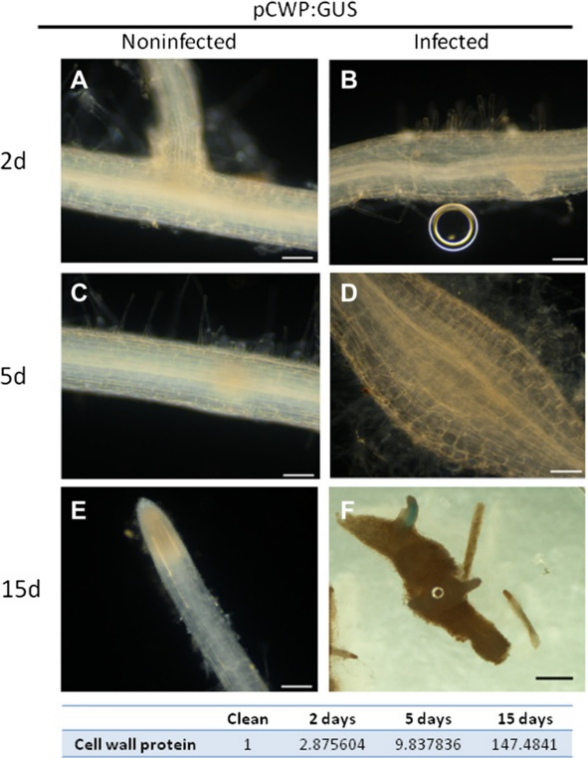
**Cell wall protein (CWP) promoter (pCWP)–GUS expression in transgenic tomato hairy root line infected with** ***M. javanica*** **second-stage juveniles (J2s).** Noninfected control **(A, C)** and infected **(B, D)** roots harboring the pCWP–GUS fusion construct show no GUS staining of the root or lateral root at 2 and 5 DAI. At 15 DAI, no signal is observed in noninfected roots **(A, C, E)**. Although infected roots at 2 and 5 DAI **(B, D)** showed no signal, GUS signal is observed in lateral roots associated with galls induced by the invading nematodes at 15 DAI **(F)**. **(A–E)** Light micrographs as viewed under a light microscope. **(F)** Bright-field image of galls photographed using a stereomicroscope. Bars: **A–E** = 100 μm, **F** = 1000 μm. Differential expression of CWP in OE roots compared with Kan roots obtained in the RNA-Seq data is shown at the bottom of the table.

To further validate the spatial and temporal expression patterns of cell wall-related genes, we used the gene encoding CWP (Solyc09g097770.2.1) in the aforementioned promoter–GUS construct assay. In noninoculated roots of the CWP promoter–GUS hairy root line (pCWP–GUS), no CWP was detected at any of the tested time points (Figure 8A,C,E). Interestingly, after inoculation, the lateral roots adjacent to the galls showed strong signal, which indicated induction of CWP by nematode infection (Figure 8F). A similar phenotype was observed for all pCWP–GUS transformed root events.

### Transcriptome changes in the phenylpropanoid and phenylalanine pathways associated with *mj-far-1* overexpression

**Figure 9:**
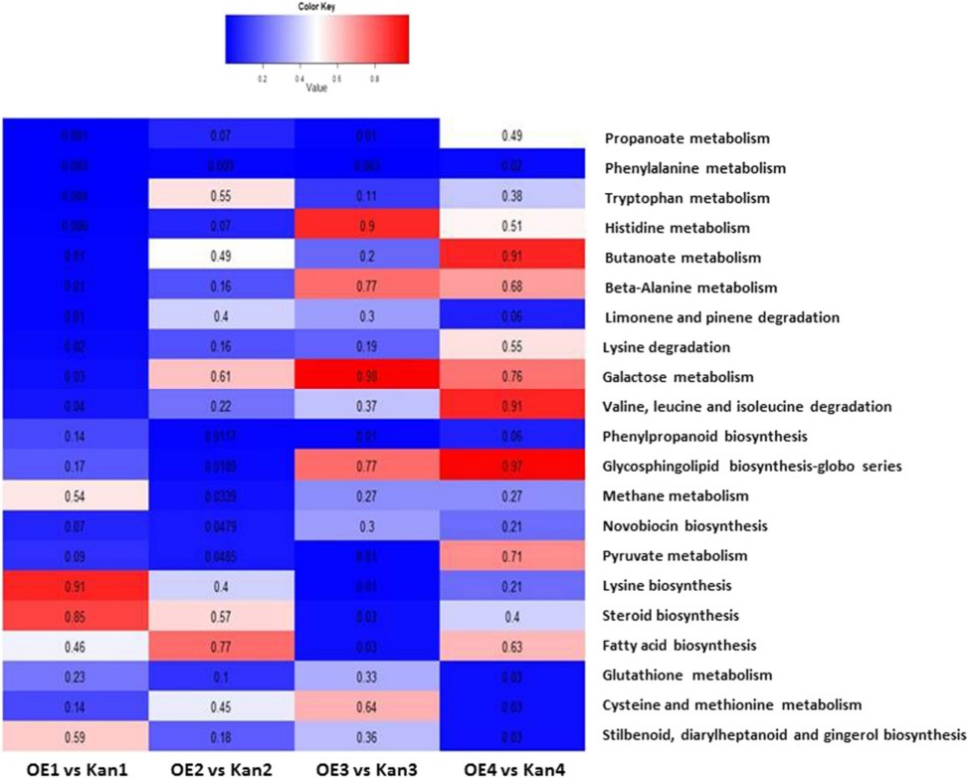
**Enriched KEGG pathway at different time points.** All 3970 differentially expressed genes were assigned to a pathway according to the KEGG database. All Kan roots are root samples of vector 11.5 carrying the kanamycin-resistance gene (Kan1, Kan2, Kan3, and Kan4 refer to Kan control roots that were noninoculated and at 2, 5, and 15 DAI, respectively) and OE roots are *mj-far-1.1* lines overexpressing *mj-far-1* (OE1, OE2, OE3, and OE4 refer to OE roots that were noninoculated and at 2, 5, and 15 DAI, respectively). KEGG pathway enrichment at each time point was calculated using the hypergeometric test. The *P*-values are presented on the heat map: the color gradient from dark blue to red represents strongly and significantly enriched pathways to nonsignificantly enriched pathways, respectively.

**Figure 10:**
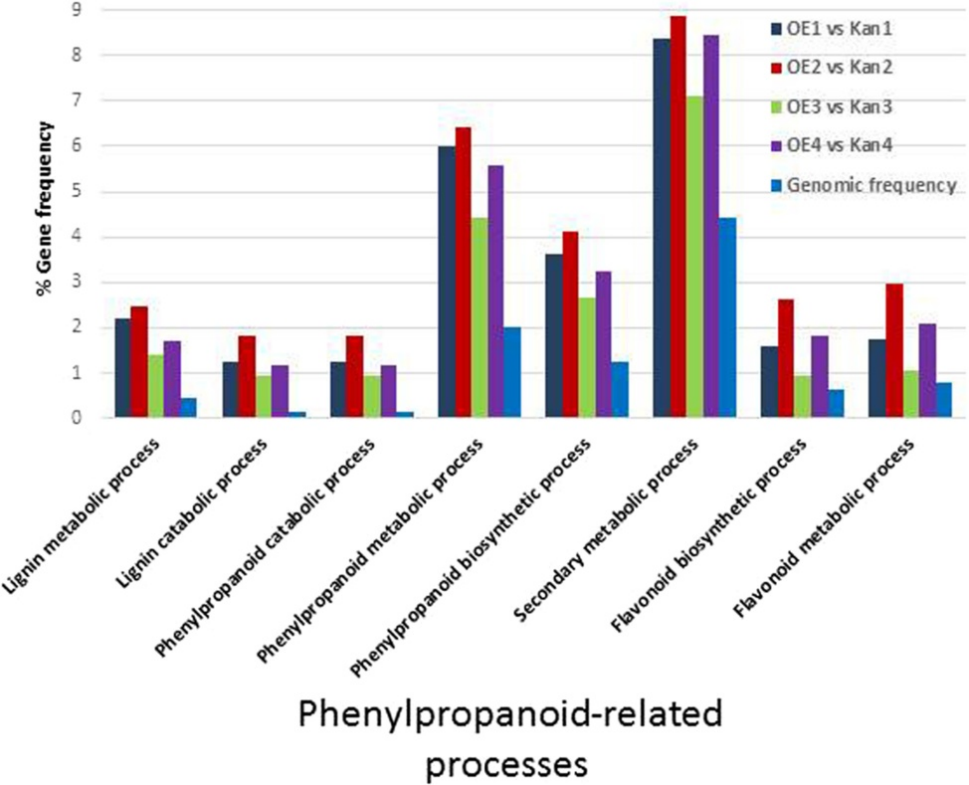
**Frequency distribution of reads putatively associated with phenylpropanoid processes identified among differentially expressed genes (DEGs) from root transcriptomes of all noninfected and infected samples.** All Kan roots are root samples of vector 11.5 carrying the kanamycin-resistance gene (Kan1, Kan2, Kan3, and Kan4 refer to Kan control roots that were noninoculated and at 2, 5, and 15 DAI, respectively) and OE roots are *mj-far-1.1* lines overexpressing *mj-far-1* (OE1, OE2, OE3, and OE4 refer to OE roots that were noninoculated and at 2, 5, and 15 DAI, respectively). Each category of secondary metabolite-related genes is indicated on the *x*-axis and the percentage of genes from each category relative to the ITAG2.3 reference tomato genome and to the DEGs is indicated on the *y*-axis.

All 3970 DEGs were annotated using the Kyoto Encyclopedia of Genes and Genomes (KEGG) database [39] for pathway-enrichment analysis relative to the available whole transcriptome annotation for tomato (ITAG2.3), using the hypergeometric test. Enrichment of the different pathways at each time point is summarized in Figure 9. Pathway-enrichment analysis revealed the predominance of both phenylalanine and phenylpropanoid pathways in roots overexpressing *mj-far-1* compared with Kan roots at all tested time points (Figure 9). This pattern was demonstrated by a high representation of genes belonging to the secondary metabolism subcategories (Figure 10), whereby transcripts belonging to certain subcategories were overrepresented in the DEGs at specific time points compared with their frequency in the tomato genome.

The phenylpropanoid pathway leads to the synthesis of coumarins, flavonoids, phytoalexins, lignins, and lignans, all of which can contribute to plant defense. In roots overexpressing *mj-far-1*, decreased levels of a transcript similar to phenylalanine ammonia-lyase (PAL) 2 (Solyc03g042560.1.1) and a transcript similar to 4-coumarate-CoA ligase (4CL3) (Solyc03g097030.2.1) were observed before nematode inoculation. The latter protein has a pivotal role in the biosynthesis of plant secondary compounds at the divergence point from general phenylpropanoid metabolism to several major branch pathways [40]. At 15 DAI, upregulation of two transcripts similar to PAL1 (Solyc00g282510.1.1 and Solyc10g011930.1.1) and a transcript similar to PAL4 (Solyc03g036480.1.1) by 3.3-, 4.18- and 2.82-fold, respectively, was observed. Transcripts similar to cinnamyl alcohol dehydrogenase 9 (CAD9) (Solyc08g014360.1.1 and Solyc02g069250.2.1), a key enzyme in lignin biosynthesis [41], were strongly upregulated (553.4- and 2.19-fold, respectively) in roots overexpressing *mj-far-1* at 2 DAI. Similarly, expression of the gene encoding CHS (Solyc05g053550.2.1), a key enzyme in flavonoids biosynthesis, was differentially regulated in all inoculated OE vs. Kan roots (Table 3), showing increased transcript levels at 2 and 5 DAI followed by a decreased transcript level at 15 DAI.

### Quantitative reverse-transcription (qRT)-PCR validation of RNA-Seq data

To confirm the expression profiles obtained from the RNA-Seq data, qRT-PCR analysis was carried out for 22 genes selected from among the 61 and 52 common DEGs in OE roots compared with Kan roots for overall noninoculated and inoculated samples, and for inoculated-only samples, respectively (Tables 2 and 3). Quantitative RT-PCR was performed using RNA isolated from infected and noninfected root samples of both root lines from the same batch used for preparation of the whole root transcriptome (Additional file 3: Table A3). The genes were selected to represent both up- and downregulated genes with log2 changes ranging from 3.11-fold upregulation to 12.08-fold downregulation in the transcriptome analysis. Of the 22 genes tested, 19 (86.3%) showed differential expression in the direction observed in the transcriptome profiling (Additional file 3: Table A3). For example, we confirmed the constant downregulation of the genes encoding long-chain fatty acid-CoA ligase (Solyc08g008310.2.1) and indole-3-acetic acid-amido synthetase (Solyc02g092820.2.1), together with constant upregulation of the genes encoding nodulin family protein (Solyc05g055540.1.1) and chitinase (Solyc07g009510.1.1). Only two probe sets that were shown to be downregulated in the RNA-Seq analysis, namely esterase/lipase/thioesterase (Solyc05g018770.1.1) and auxin-responsive protein (Solyc08g021820.2.1), were slightly upregulated in the qRT-PCR analysis at 5 and 15 DAI. Similarly, the gene encoding the fatty acid elongase 3-ketoacyl-CoA synthase (Solyc03g005320.2.1), which was shown to be upregulated in the RNA-Seq analysis, was downregulated in the qRT-PCR analysis at 5 and 15 DAI (Additional file 3: Table A3). Thus, overall, the qRT-PCR and RNA-Seq results were in agreement.

## Discussion

Following deposition of effectors by the nematode through the stylet [5-9] or other organs in contact with the external environment, such as the amphids or cuticle [4,12,13], the question of how these proteins ultimately perform their function in the host cells remains to be answered. This is a complex aspect of plant–nematode interactions that are being dissected by several research groups [42]. A range of functions are attributed to nematode effectors, apart from reprogramming cell metabolism for the generation and maintenance of the nematodes’ feeding sites. A relatively large subset of effectors deals with the suppression of defense responses triggered by parasitism [42,43]. Data to date indicate that, as with other pathogens, active suppression of host defense responses is a critical component of successful parasitism by nematodes [42].

The involvement of nematode effectors in promoting host susceptibility to nematode infection has been reported for the cyst nematode *Heterodera* effectors CBP, 10A06, 4FO1, and 30CO2, and for the RKN *Meloidogyne incognita* effector CRT, whose overexpression in *Arabidopsis* increases susceptibility to nematodes [27,28,42-45]. Similarly, studies of the FAR protein from *M. javanica* (Mj-FAR-1) have shown that tomato hairy roots overexpressing *mj-far-1* are remarkably more susceptible to RKNs [26]. The observed increase in host susceptibility conferred by successful pathogens could be the result of their overcoming pattern-triggered immunity, via suppression of the pattern-triggered immunity response by secreted effectors, leading to effector-triggered susceptibility (ETS) [42]. Even though the function of nematode infection in the manipulation of plant defense has been extensively studied, it is clear that nematodes target different levels of the plant’s immune system.

In this study, we aimed to elucidate the contribution of the protein Mj-FAR-1, which is secreted to suppress plant defense responses and to promote ETS, by exploring the broad transcriptional events underlying the increased susceptibility observed in roots overexpressing *mj-far-1* relative to control roots. Using the RNA-Seq approach, we observed that *mj-far-1* overexpression accounts for the differential expression of 3970 transcripts before and after inoculation. Of these transcripts, 2069 were upregulated and 2205 were downregulated in the OE vs. Kan roots for all inoculated and noninoculated samples. This finding is in agreement with previous results in which nematode infection induced not only upregulation of the transcription of specific enzymes, but also downregulation of transcriptional, translational, and catalytic events [14]. Collectively, these results suggest that the nematode might manipulate multiple pathways to suppress the host defense response. Pearson correlation tests indicated that samples of noninoculated roots and roots in the first phase of parasitism, i.e., prior to feeding (2 DAI) when the nematode is initiating attempts to establish itself in the host, clustered predominantly according to the infection step*.* However, at 5 and 15 DAI, during feeding-site formation and execution of the compatibility response, *mj-far-1* overexpression had a greater impact on defining the root transcriptome of OE and Kan lines (Figure 3A). These results support our transcriptomic data, as global changes occurring in response to nematode infection are predicted to be similar in both lines, but the number and response level of modulated genes should provide a global overview of the changes that are attributable to Mj-FAR-1.

### Regulation of hormone signaling-related genes by *mj-far-1*

Examination of genes encoding host biochemical pathways that have been implicated in the response to RKNs identified several hormone pathways that might be subject to *mj-far-1* manipulation. The observed downregulation of JAZ1 together with upregulation of AOS in noninoculated roots expressing *mj-far-1* might induce the JA pathway and support nematode invasion and establishment in the first stages of infection. Furthermore, the observed upregulation of Ethylene Response Factor 1 might indicate that JA levels are increased because this gene is known to be activated by both JA and ET [46,47]. Coincident with the suggested upregulation of the JA pathway in noninoculated OE roots, a reduction in PAL levels was indicated. These observations might support an antagonistic interaction between SA and JA in OE roots [48-50]. Moreover, upregulation of salicylic acid carboxyl methyltransferase (solyc09g091550.2.1) at 2 DAI might indicate a decrease in SA accumulation. This suggestion is supported by previous findings in which overexpression of salicylic acid carboxyl methyltransferase reduces SA-mediated pathogen resistance in *Arabidopsis thaliana* [51]. Evidence for the possible role of JA in promoting nematode development has been reported by Bhattarai et al. [52], who analyzed tomato mutants altered in JA signaling and concluded that an intact JA-signaling pathway is required for tomato susceptibility to RKNs. Similarly, in maize, Mu-insertional *lox3-4* mutants displayed increased attractiveness to RKNs, and an increased number of juveniles and eggs were accompanied by elevated levels of JA [53]. More recently, in *Arabidopsis*, the 13-LOX member, *lox4-1* mutant, characterized by increased levels of JA, demonstrated increased susceptibility to RKNs [54]. Overall, these results support our findings of increased JA signal promoting root susceptibility to RKNs. Following inoculation, several transcripts similar to *Arabidopsis* 9- and 13-LOX were differentially regulated in roots overexpressing *mj-far-1* relative to control roots. LOXs are widely present in higher plants; they are important enzymes in the biosynthesis of oxylipins and in the plant response to wounding and pathogen attack [55]. Although only the 13-LOX pathway has been implicated in JA biosynthesis, other studies have suggested that there may be another as-yet-unknown pathway leading to LOX-mediated defense responses [56]. The observed fluctuation in the expression patterns of LOX-encoding transcripts suggests that nematode development requires the dynamic coordinated expression of LOX genes in the proper order for successful establishment in a susceptible root. The altered expression of LOX genes as a result of Mj-FAR-1 in OE compared with Kan roots is supported by results from an *in vitro* study indicating that LOX activity was inhibited by FAR-1 of the potato nematode *G. pallida* [11]. Thus, lipid-binding activity of Mj-FAR-1 toward free fatty acids which, among others, are LOX substrates might be involved in manipulating the plant defense response mediated by fatty acid signaling.

Downregulation of several auxin-related genes was observed in OE vs. Kan control roots, including those genes encoding indole-3-acetic acid-amido synthetase (GH3.8) and auxin-responsive GH3-like, which were downregulated in all OE root samples (Additional file 1: Table A1). These results are consistent with the hypothesis that, in general, global alterations of auxin balance accompany RKN infection [57]. Moreover, the finding that all transcripts similar to JAZ1 were downregulated at later time points after infection suggests that strict regulation of JAZ1 is an infection strategy that enables nematode development. Recent evidence indicating that *jaz1* is not only a JA-responsive but also an auxin-responsive gene further illustrates the intimate molecular interplay between auxin and JA signaling [58]. Collectively, these results suggest that Mj-FAR-1 is involved in manipulating multiple pathways to coordinate feeding-site formation, protection from host defense responses, and maintenance of GCs.

### Regulation of cell wall organization-related genes by *mj-far-1*

In the present study, strong representation of DEGs associated with cell wall-related activities was detected. The extensive modifications in cell wall architecture (i.e., thickening, ingrowth, disassembly, and dissolution) that occur in cyst nematode and RKN feeding cells are likely mediated by the activity of both cell wall-biosynthetic and cell wall-degrading enzymes. Interestingly, most of the cell wall biosynthesis-, organization-, and modification-related genes were downregulated (more than 3-fold) in OE vs. Kan roots, and particularly in noninoculated roots at 2 and 5 DAI. These data further confirm the hypothesis that cell wall biosynthesis, modification or fortification is essential to the plant’s response to nematode infection. Similar to other transcriptomic studies, RNA-Seq data indicated that the expression of many genes involved in cell wall extension and remodeling is altered following nematode inoculation [59-61]. For example, a group of genes involved in cellulose synthesis was downregulated at an early time point, whereas several transcripts were upregulated at 15 DAI in OE vs. Kan control roots (Figure 7C). Cellulose synthesis is expressed in the initial expansion phase of GC development. Concomitant hyperplasia of root cells surrounding the GCs to form the visible gall also likely requires synthesis of new (primary) cell wall [62-64]. It might be that upregulation of this group of genes at 5 and 15 DAI reflects the accelerated disease development observed on the OE root line. Similarly, there was impressive representation of expansin-encoding genes (Figure 7B). Expansins are encoded by a large multigene family. They are identified as wall-loosening factors and facilitators of cell expansion [64,65]. A previous study indicated that a decrease in tomato EXPA5 expression by means of RNAi-transgenic root generation reduces the nematode’s ability to complete its life cycle in transgenic roots [65]. Following GC formation, and possibly as a secondary response, division and expansion of cortical and pericycle cells around the GCs occur, causing the formation of galls. Similarly, specific transcripts among the group of genes encoding pectin esterases, pectate lyase and polygalacturonase were upregulated in OE vs. Kan roots at the later time points (Figure 7).

Cell expansion, cell elongation, cell wall biosynthesis, and cell wall dissolution are all physiological processes that have been observed indirectly within nematode-induced feeding cells [66,67]. The differential regulation of cell wall biosynthesis- and modification-related gene expression at the early time point might facilitate nematode establishment in root tissues.

### Regulation of phenylpropanoid-related genes by *mj-far-1*

Notable differences in the expression of genes encoding enzymes in the phenylpropanoid pathway were observed in OE vs. Kan control roots at all time points (Figure 9). Many secondary metabolites derived from multiple branches of the phenylpropanoid pathway, including lignins, isoflavonoid-phytoalexins, other phenolic compounds, and SA, are instrumental in the plant’s ability to mount a successful defense against invading pathogens [68]. A remarkable decrease in the expression of genes encoding enzymes at the initiation of the phenylpropanoid pathway, e.g., genes encoding PAL and 4CL3, was observed in noninoculated OE vs. Kan control roots. PAL (EC 4.3.1.34) can be considered a control point for entry into the phenylpropanoid pathway [69], whereas 4CL3 has a pivotal role in the biosynthesis of plant secondary compounds at the divergence point from general phenylpropanoid metabolism to several major branch pathways [38]. At 5 and 15 DAI, four genes encoding different PAL isoforms increased in expression, thereby suggesting increased metabolic flow into the phenylpropanoid pathway. Increased PAL enzyme activity has been noted in resistant tomato roots infected with RKNs, whereas PAL activity is depressed in susceptible tomato roots [70]. Similarly, in potato, PAL activity is higher in resistant plants [71]. It may be hypothesized that the decrease in PAL expression before inoculation facilitates nematode infection, whereas the high level of PAL at 5 and 15 DAI reflects acceleration of the infection progress promoted in the OE root. Two transcripts encoding CAD9 showed increased expression at 2 DAI; CAD9 is a key enzyme in lignin biosynthesis as it catalyzes the final step in the synthesis of monolignols. Its expression may be the result of increased penetration and accelerated disease progression in the OE line (Solyc02g069250.2.1 and Solyc08g014360.1.1, with 2.19- and 553.4-fold increases in expression, respectively). An additional important gene is CHS, which was upregulated at 2 and 5 DAI and downregulated at 15 DAI. CHS is involved in glyceollin synthesis, which is known to inhibit oxygen uptake by *Meloidogyne* [72]. The decrease in CHS expression at 15 DAI might support nematode infection. Invasion of roots with RKNs and cyst nematodes induces the flavonoid pathway in infection structures [57,73], and flavonoids are hypothesized to act as regulators of auxin transport and accumulation during gall formation [57,58]. In flavonoid-deficient *Medicago truncatula* plants, gall formation still occurred, although galls were smaller and showed fewer cell divisions [74]. In flavonoid-deficient *Arabidopsis* and tobacco mutants, reproduction of several species of nematodes was not affected [73,75]. However, flavonoids did affect nematode behavior; for example, certain flavonoids acted as repellents for specific nematode species and inhibited their motility and hatching at millimolar concentrations [76]. Although synthesis of flavonoid or isoflavonoid phytoalexins, deposition of lignin or cell wall-bound phenolics, and synthesis of other defense chemicals via the phenylpropanoid pathway are often characteristic of both the localized hypersensitive response and systemic acquired resistance [77], we suggest that in OE roots, decreased abundance of transcripts associated with synthesis and regulation of defense chemicals derived from the phenylpropanoid pathway might facilitate nematode infection.

## Conclusions

The present study provides evidence for the potential mediation by Mj-FAR-1 of a complex defense-related response, including differential regulation of cell wall-, hormone- and fatty acid-related genes, as well as changes in the phenylpropanoid pathway. Our results indicate that roots overexpressing *mj-far-1* still mount a defense response against nematode infection; however, this rapid response might reflect the accelerated disease progress in OE roots upon nematode infection. While the direct effects of *mj-far-1* might be related only to fatty acid metabolism, the indirect effect mediated by lipid signaling may drive other pathways that affect plant responses to nematodes. This study adds to our understanding of the role of *mj-far-1* and may ultimately indicate novel pathways that are required for nematode establishment and parasitism.

## Methods

### Plant materials and growth conditions

Tomato ‘Avigail’ )870) was used as the background line for both transgenic root lines: *mj-far-1* OE and the control Kan, as described previously [26]. Both root lines were subcultured on standard-strength Gamborg’s B5 salt medium (Duchefa, Haarlem, The Netherlands), supplemented with 2% (w/v) sucrose and solidified with 0.8% (w/v) Gelrite agar (Duchefa). Roots were subcultured on B5 medium, with one root section per petri dish (Miniplast, Ein Shemer, Israel), and incubated horizontally in a growth chamber at 26°C in the dark for 1 week to allow root branching before nematode inoculation.

### Nematode culture and infection assays

*Meloidogyne javanica* was propagated on greenhouse-grown tomato ‘Avigail’ (870) plants. Nematode egg masses were extracted from roots with 0.05% (v/v) sodium hypochlorite followed by sucrose flotation [78]. For sterilization, eggs were placed on a cellulose–acetate filter membrane (Sartorius Stedim Biotech GmbH, Goettingen, Germany, pore size 5 μm) in a sterile Whatman® filter holder (Whatman International Ltd., Dassel, Germany). Eggs on the filter were exposed for 10 min to 0.01% (w/v) mercuric chloride (Sigma-Aldrich, St Louis, MO, USA), followed by 0.7% (v/v) streptomycin solution (Sigma-Aldrich), and three washing steps with 50 ml sterilized distilled water [79]. The sterilized eggs were collected from the membrane and placed on 25-μm-pore sieves in 0.01 M 2-morpholinoethanesulfonic acid buffer (Sigma-Aldrich) under aseptic dark conditions for 3 days, allowing J2s to hatch. Freshly hatched preparasitic J2s were collected in a 50 ml falcon tube. For nematode infection, 1-week-old transgenic tomato root lines, growing on standard-strength Gamborg’s B5 salt medium, were inoculated with 200 sterile freshly hatched *M. javanica* preparasitic J2s. Plates were left uncovered in a laminar flow hood until water had completely soaked into the medium [80]. The inoculated and noninoculated roots were incubated horizontally in the dark, and root samples were taken for either RNA extraction or GUS bioassay at the designated time points after inoculation.

### cDNA library preparation and high-throughput sequencing

Total RNA was extracted using TRI reagent (Sigma-Aldrich) from Kan and OE tomato root lines at different time points postinoculation. Beads containing oligo (dT) were used to isolate poly(A) mRNA from 500 μg total RNA for each sample. Purified mRNA was then fragmented in fragmentation buffer. Using these short fragments as templates, random hexamer primers were used to synthesize the first-strand cDNA. The second-strand cDNA was synthesized using buffer, dNTPs, RNase H and DNA polymerase I. Short double-stranded cDNA fragments were purified with the QIAquick PCR Purification Kit (QIAGEN Inc., Valencia, CA) and eluted with elution buffer (EB) for end repair and the addition of an ‘A’ base. The short fragments were ligated to Illumina sequencing adaptors. DNA fragments of a selected size were gel-purified and amplified by PCR. The amplified library was sequenced on an Illumina HiSeq™ 2000 platform. The details of the experiment were as follows: expected library size, 200 bp; read length, 90 nucleotides; sequencing strategy, paired-end sequencing. The library size and read length are provided in the Additional file 1: Table A1.

### Read alignment to the reference tomato genome

In total, 212,975,340 2 × 100 bp reads were sequenced. Read mapping to the ITAG *Solanum lycopersicum* protein reference version 2.3 (ITAG2.3; http://solgenomics.net) was performed with SoapAligner/SOAP2 [29]. An average of 20.1 million reads from each library paired-end sequencing were uniquely aligned to the reference sample, and overall made up ca. 75.6% of the total reads (Table 1) used in the bioinformatics analysis. Gene-expression level was normalized using the RPKM (reads per kilobase transcriptome per million mapped reads) method [81].

Differences in gene expression between two samples were calculated based on Poisson distribution for gene expression. FDR was calculated using the Benjamini and Yekutieli (2001) FDR method [82]. We used FDR ≤ 0.001 and the absolute value of log2 ratio ≥ 1 as the thresholds to judge the significance of the differences in gene expression. All sequences were uploaded to the NCBI SRA database under accession no. SRX504894.

Differences in gene expression were visualized using MapMan [31,83]. The MapMan mapping file was obtained from http://www.gomapman.org/; 27,212 of the 29,549 genes on the microarray were present in the mapping file. Enrichments of functional categories of the MapMan annotation in the significantly DEGs were tested for significance by applying Fisher’s test with Bonferroni correction for multiple tests using Mefisto Version 0.23beta (http://www.usadellab.org). Enrichment of Gene Ontology (GO) terms in significantly DEGs was evaluated using the agriGO GO analysis toolkit (http://bioinfo.cau.edu.cn/agriGO) [84] with Fisher’s test and Bonferroni multiple testing correction (*P* < 0.05). Pathway analysis was done using the KEGG database [38] and enrichment was calculated using the hypergeometric test followed by the FDR test. PCA analysis was performed using the FactoMineR package [29].

### Real-time qPCR analysis

For qPCR experiments, contaminant genomic DNA was removed from the RNA with the Turbo DNA-free Kit from Ambion (Applied Biosystems, Foster City, CA, USA). DNA-free RNA (1 μg) was converted into first-strand cDNA using the Verso™ cDNA Synthesis kit (ABgene, Epsom, UK), and reactions were performed using the ABsolute SYBR Green ROX mix (ABgene). Primers for qRT-PCR experiments were designed with Primer Express software (Applied Biosystems; see Additional file 4: Table A4). The real-time PCR contained 3.4 μl cDNA in a total volume of 10 μl, consisting of 1× SYBR-Green Amplification Kit (ABgene), 150 nM forward primer and 150 nM reverse primer, and was run in real-time PCR plasticware (Axygen, Union City, CA, USA). All PCR cycles began with 2 min at 50°C, then 10 min at 95°C, followed by 40 cycles of 10 s at 95°C and 1 min at 60°C. After the PCR, a melting curve was generated by gradually increasing the temperature to 95°C to test for amplicon specificity. For qPCR, a mixture of all cDNAs was used for all treatments, as a template for calibration curves designed for each pair of primers. Each reaction was performed in triplicate and the results represent the mean of two independent biological experiments. Three constitutively expressed genes, namely actin (*ACT*; GenBank accession no. U60482.1), *β-tubulin* (*TUB*; GenBank accession no. NM_001247878.1) and *18S* (GenBank accession no. BH012957.1), were used as endogenous controls for gene expression analysis (Additional file 4: Table A4). Transcript levels were normalized for each sample with the geometric mean of the corresponding selected housekeeping genes. All of the housekeeping genes were confirmed to display minimal variation across the treatment and were the most stable housekeeping genes from a set of tested genes in a given cDNA sample. Values were expressed as the increase or decrease in level relative to a calibration sample. The following control reactions were included: PCR negative control without cDNA template to confirm the absence of nonspecific PCR products (NTC), and a second reaction containing mRNA that had not been subjected to reverse transcription (NRT control). To confirm the expression profiles obtained from the RNA-Seq expression data, RT-qPCR analysis was carried out for 22 genes selected on the basis of their biological significance: genes involved in fatty acid metabolism, such as long-chain fatty acid-CoA ligase and fatty acid elongase, cell wall-related transcripts such as chitinase, expansin-1 and CWP, and hormone-related transcripts such as auxin-responsive protein and indole-3-acetic acid-amido synthetase.

### Plasmid construction and generation of transgenic tomato roots

All PCR amplifications used for plasmid construction were performed using the Dream Taq Green Master Mix (Thermo Fisher Scientific, Pittsburgh, PA, USA) in accordance with the manufacturer’s instructions and using tomato genomic DNA as the template. To clone the different promoter sequences (pLOXD and pCWP), specific primers designed to amplify a 2000 bp fragment and to create the *Sac*I and *Sma*I restriction sites at the 5′ and 3′ ends of the promoter, respectively, were used (Additional file 5: Table A5). The *Sma*I restriction site was placed before the ATG sequence of the respective genes to guarantee the correct reading frame when the promoter was fused to the β-glucuronidase (GUS) gene. The 2000 bp fragment was then cloned into the pUC19_Y vector [37] at the *Sac*I and *Sma*I restriction sites. The 4010 bp cassette containing the specific gene promoter and the GUS reporter gene was then isolated by restriction digestion with *Sac*I and *Sal*I and subsequently cloned into the pCAMBIA2300 binary vector [38]. The identity, orientation, and junctions of the resulting pCAM-LOXD and pCAM-CWP constructs were confirmed by digestion patterns and sequencing. Five different events of transformed roots with pCAM-LOXD or pCAM-CWP were subjected to the GUS assay, with 10 specimens sampled for each root line. A supplementary plasmid pME-524, expressing GUS under the control of the 35S promoter, was used as a positive control. The pCAMBIA2300 empty-vector control and the construct plasmids were subsequently used for *A. rhizogenes*-mediated root transformation.

### *Agrobacterium-*mediated root transformation and production of hairy root cultures

The binary vector pCAM-LOXD, pCAM-CWP and the empty-vector control pCAMBIA2300 were electrotransformed into *A. rhizogenes* ATCC 15834 [85]. Individual cotyledons were excised from 8–10-day-old tomato seedlings and immersed in an *A. rhizogenes* suspension (OD_600_ 1.0) for 15 min. The excised cotyledons were blot-dried on sterile filter paper, then co-cultivated on standard-strength Gamborg’s B5 salt medium for 3 days. Explants were then washed with liquid B5 medium supplemented with the antibiotics kanamycin (50 μg ml^−1^) (Duchefa Biochemie) and Timentin (300 μg ml^−1^; ticarcillin disodium:potassium clavulanate, 15:1) (Duchefa Biochemie) and incubated at room temperature for 1 h with mild shaking. The explants were blot-dried on sterile filter paper and placed on B5 agar medium supplemented with the same antibiotics. Within 7 to 10 days of incubation at 25°C in the light, roots emerged on the surface of the cotyledons. Hairy roots were transferred to Gamborg’s B5 medium supplemented with 0.8% (w/v) Gelrite and kanamycin (50 μg ml^−1^).

### Histochemical localization of GUS activity and microscopic analysis

One-week-old control and promoter-GUS tomato roots were inoculated as described above, and assayed histochemically for GUS activity at 2, 5 and 15 DAI. A set of noninfected plates served as the control group. For GUS assays, infected and noninfected transgenic root tissues were removed from the petri dishes at specific time points after inoculation and infiltrated with GUS-staining buffer containing 50 mM sodium phosphate (pH 7.0), 10 mM EDTA, 5 mM K_4_[Fe_2_(CN)_6_], 5 mM K_3_[Fe_2_(CN)_6_], 0.2% (v/v) Triton X-100 and 2 mM 5-bromo-4-chloro-3-indolyl ß-D-glucuronide (X-Gluc). GUS staining was performed for 12 h at 37°C. For observation and documentation, GUS-stained roots were mounted on microscope slides or in small wells, and photographed with either a Leica DMLB light microscope and a Nikon Eclipse 90i (Leica Microsystems GmbH, Wetzlar, Germany; Nikon Corporation, Tokyo, Japan), or a stereomicroscope (Leica MZFLIII, Leica Microsystems GmbH) equipped with a Nikon DS-Fi1 camera.

### Availability of supporting data

All the referred supporting data are included as additional files.

## Additional files

Additional file 1: Table A1. Differentially expressed genes related to biotic stress as represented in the MapMan illustration. Additional file 2: Table A2. Differentially expressed genes involved in cell wall metabolism. Additional file 3: Table A3. Validation of RNA-Seq results by qRT-PCR. Additional file 4: Table A4. Forward and reverse primer sequences used for qRT-PCR confirmation of expression levels of differentially expressed genes from the transcriptomic data, and primers sets for internal control genes used to normalize gene expression in the qRT-PCR analysis. Additional file 5: Table A5. Primer pairs used for amplification of promoter fragments.
