# Supplementary material for: Meloidogyne javanica fatty acid- and retinol-binding protein (Mj-FAR-1) regulates expression of lipid-, cell wall-, stress- and phenylpropanoid-related genes during nematode infection of tomato
Source: BMC Genomics. 2015 Apr 8;16(1):272. doi: 10.1186/s12864-015-1426-3 (PMC4450471; doi:10.1186/s12864-015-1426-3)
Supplement: Additional file 1: Table A1. — Differentially expressed genes related to biotic stress as represented in the MapMan illustration. [file 12864_2015_1426_MOESM1_ESM.pdf]

**Table A1.** The list of DEG belongs to biotic stress represented in MapMan illustration.

| *Functional categories* | *sub-category* | *Gene ID* | *OE2-KAN2* | *Annotation* |
|---|---|---|---|---|
| Cell wall | Precursor synthesis | solyc06g069550.1.1 | -2.11955 | UDP-D-glucose dehydrogenase |
| | | solyc05g054590.2.1 | -2.05549 | Bifunctional polymyxin resistance protein ArnA |
| | | solyc08g069110.2.1 | -7.29577 | Dtdp-glucose 4 6-dehydratase |
| | | solyc05g046340.1.1 | -835.908 | Phosphomannomutase 3 |
| | Cellulose synthesis | solyc09g009010.2.1 | -2.07662 | Cellulose synthase-like C1-2 glycosyltransferase family 2 protein |
| | | solyc02g072240.2.1 | -2.18779 | Cellulose synthase |
| | | solyc07g005840.2.1 | -2.10016 | Cellulose synthase 3 |
| | | solyc09g072820.2.1 | -2.43029 | Cellulose synthase |
| | | solyc03g070440.2.1 | -2.22474 | COBRA-like protein |
| | | solyc03g114900.2.1 | -2.2787 | COBRA-like protein |
| | | solyc09g075540.1.1 | -2.37443 | COBRA-like protein |
| | Cell wall proteins | solyc09g007650.1.1 | -3.93894 | Fasciclin-like arabinogalactan protein 7 |
| | | solyc06g075220.1.1 | -2.26688 | Fasciclin-like arabinogalactan protein 5 |
| | | solyc09g007660.1.1 | -2.98588 | Fasciclin-like arabinogalactan protein 7 |
| | | solyc11g069250.1.1 | -2.01137 | Fasciclin-like arabinogalactan protein |
| | | solyc07g053840.1.1 | -4.58789 | LRR receptor-like serine/threonine-protein kinase, RLP |
| | Degradation | solyc03g083820.1.1 | -2.02833 | Endoglucanase 1 |
| | | solyc08g081620.2.1 | -589.422 | Endoglucanase 1 |
| | | solyc01g111340.2.1 | -2.26191 | Endo-1 4-beta-xylanase |
| | | solyc01g079570.2.1 | -2.25082 | Beta xylosidase |
| | | solyc11g040330.1.1 | -2.16596 | Endo-1 4-beta-xylanase |
| | | solyc05g005040.2.1 | -3.80312 | Polygalacturonase |
| | | solyc12g019120.1.1 | -5.15505 | Polygalacturonase |
| | | solyc12g096750.1.1 | -10.3227 | Polygalacturonase 4 |
| | | solyc04g025440.2.1 | -2.42823 | Polygalacturonase |
| | | solyc05g014000.2.1 | -2.36055 | Pectate lyase |
| | | solyc03g114240.2.1 | -2.02939 | BURP domain-containing protein (Fragment) |
| | | solyc12g019130.1.1 | -3.95129 | Polygalacturonase |
| | | solyc06g068040.2.1 | -15.2125 | Polygalacturonase |
| | | solyc12g019230.1.1 | -4.34642 | Polygalacturonase 1 |
| | | solyc08g060970.2.1 | -2.3641 | Polygalacturonase |
| | | solyc08g068150.2.1 | -2.26084 | BURP domain-containing protein |
| | | solyc12g019140.1.1 | -3.53724 | Polygalacturonase |
| | | solyc02g062300.2.1 | -858.592 | BURP domain-containing protein |
| | Modification | solyc01g090810.2.1 | -2.09576 | Expansin protein |
| | | solyc08g077900.2.1 | -2.97689 | Expansin-like protein |
| | | solyc12g011030.1.1 | -2.54949 | Xyloglucan endotransglucosylase/hydrolase 7 |
| | | solyc10g005350.2.1 | -2.78442 | Xyloglucan endotransglucosylase/hydrolase 1 |
| | | solyc07g009380.2.1 | -2.24973 | Xyloglucan endotransglucosylase/hydrolase 2 |
| | | solyc10g086520.1.1 | -2.8156 | Expansin-1 |
| | | solyc03g093390.2.1 | -2.35591 | Expansin protein |
| | | solyc10g084780.1.1 | -3.97387 | Expansin |
| | | solyc07g006860.2.1 | 3.374426 | Xyloglucan endotransglucosylase/hydrolase 3 |
| | | solyc03g098430.2.1 | -2.55672 | Xyloglucan endotransglucosylase/hydrolase 5 |
| | | solyc07g006870.2.1 | -2.41173 | Xyloglucan endotransglucosylase/hydrolase 8 |
| | | solyc04g081870.2.1 | -2.07729 | Expansin |
| | | solyc12g007260.1.1 | -2.45414 | Xyloglucan endotransglucosylase/hydrolase 2 |
| | | solyc12g089380.1.1 | -2.01632 | Expansin |
| | | solyc09g010860.2.1 | -2.04627 | Expansin |
| | Pectin*esterases | solyc12g008530.1.1 | -2.23358 | Pectinesterase |

| | | | | |
|---|---|---|---|---|
| | | solyc04g080530.2.1 | -9.59834 | Pectinesterase family protein |
| | | solyc03g083870.2.1 | -2.32474 | Pectinesterase |
| | | solyc05g052540.1.1 | -2.06455 | Os03g0291800 protein (Fragment) |
| | | solyc01g079180.2.1 | -3.86348 | Pectinesterase |
| Hormone metabolism | Auxins | solyc06g059730.1.1 | -2.83725 | Auxin efflux carrier |
| | | solyc10g009480.2.1 | -3.46104 | Dopamine beta-monooxygenase |
| | | solyc12g005310.1.1 | -2.26529 | Auxin-responsive GH3-like |
| | | solyc05g056430.1.1 | -3.48857 | Auxin responsive SAUR protein |
| | | solyc12g017880.1.1 | -5.26585 | Dopamine beta-monooxygenase |
| | | solyc03g082520.1.1 | 2.059969 | Auxin responsive SAUR protein |
| | | solyc02g078500.2.1 | 2.251941 | Aluminum-induced protein-like (AHRD V1 ***-Q9FG81_ARATH)" |
| | | solyc09g097890.2.1 | -2.42675 | Membrane protein |
| | | solyc07g008290.2.1 | -2.56128 | Growth regulator like protein |
| | | solyc07g048050.1.1 | -2.16153 | Dopamine beta-monooxygenase |
| | | solyc02g092820.2.1 | -3.48534 | Indole-3-acetic acid-amido synthetase GH3.8 |
| | Brassinosteroid | solyc11g006300.1.1 | -3.05859 | 3-oxo-5-alpha-steroid 4-dehydrogenase family protein |
| | Abscisic acid | solyc08g066650.2.1 | -2.58612 | Carotenoid cleavage dioxygenase 8 |
| | | solyc11g071580.1.1 | -2.38489 | Aldehyde oxidase |
| | | solyc07g056570.1.1 | -2.31292 | 9-cis-epoxycarotenoid dioxygenase |
| | | solyc05g007300.2.1 | -2.83089 | Receptor expression-enhancing protein 5 |
| | Ethylene | solyc11g072120.1.1 | -2.74013 | 2-oxoglutarate-dependent dioxygenase |
| | | solyc11g010400.1.1 | -2.58598 | 1-aminocyclopropane-1-carboxylate oxidase |
| | | solyc09g089710.2.1 | -1072.17 | 1-aminocyclopropane-1-carboxylate oxidase-like protein |
| | | solyc02g070080.2.1 | -2.66867 | 1-aminocyclopropane-1-carboxylate oxidase 1 |
| | | solyc06g073580.2.1 | -2.83815 | 1-aminocyclopropane-1-carboxylate oxidase 1 |
| | | solyc07g061720.2.1 | 2.248512 | Gibberellin 2-oxidase |
| | | solyc11g010410.1.1 | -2.01935 | 1-aminocyclopropane-1-carboxylate oxidase |
| | | solyc03g116290.1.1 | -2.2299 | Gibberellin 2-beta-dioxygenase 2 |
| | | solyc06g060070.2.1 | -2.007 | 1-aminocyclopropane-1-carboxylate oxidase |
| | | solyc11g072310.1.1 | 2.739978 | Gibberellin 20-oxidase-3 |
| | | solyc03g116280.2.1 | -2.55878 | 1-aminocyclopropane-1-carboxylate oxidase |
| | | solyc06g073080.2.1 | 5.337729 | Flavonol synthase/flavanone 3-hydroxylase |
| | | solyc11g072200.1.1 | -2.28355 | 1-aminocyclopropane-1-carboxylate oxidase 3 |
| | | solyc02g080120.1.1 | -13.0393 | Gibberellin 2-beta-dioxygenase 7 |
| | | solyc09g010000.2.1 | 3.472034 | 1-aminocyclopropane-1-carboxylate oxidase-like protein |
| | | solyc02g036350.2.1 | -2.03712 | 1-aminocyclopropane-1-carboxylate oxidase |
| | | solyc01g093980.2.1 | -693.752 | Gibberellin 20-oxidase 4 |
| | | solyc07g026900.1.1 | -2.71651 | 1-aminocyclopropane-1-carboxylate synthase |
| | | solyc12g008740.1.1 | -2.22579 | 1-aminocyclopropane-1-carboxylate synthase |
| | | solyc02g082060.1.1 | 2.450561 | PPPDE peptidase domain-containing protein 1 |
| | | solyc03g093610.1.1 | 2.07657 | Ethylene responsive transcription factor 1b |
| | | solyc10g079230.1.1 | -13.5826 | CM0545.450.nc protein |
| | | solyc01g107490.1.1 | -16.2991 | BHLH transcription factor |
| | | solyc04g080710.2.1 | -2.17321 | BHLH transcription factor-like protein |
| | Salicylic acid | solyc09g091550.2.1 | 6.344305 | Salicylic acid carboxyl methyltransferase |
| | | solyc03g007390.2.1 | -2.40197 | Pentatricopeptide repeat-containing protein |
| | | solyc02g086230.1.1 | -3.86348 | Pentatricopeptide repeat-containing protein |
| | Jasmonate | solyc01g099180.2.1 | -360.055 | Lipoxygenase |
| | | solyc04g079730.1.1 | -4.43697 | cytochrome P450 |
