# Supplementary material for: Meloidogyne javanica fatty acid- and retinol-binding protein (Mj-FAR-1) regulates expression of lipid-, cell wall-, stress- and phenylpropanoid-related genes during nematode infection of tomato
Source: BMC Genomics. 2015 Apr 8;16(1):272. doi: 10.1186/s12864-015-1426-3 (PMC4450471; doi:10.1186/s12864-015-1426-3)
Supplement: Additional file 2: Table A2. — Differentially expressed genes involved in cell wall metabolism. [file 12864_2015_1426_MOESM2_ESM.pdf]

Table A2. Detailed list of differentially expressed genes involved in the cell wall metabolism.

Pectin Esterase		
Sample	***Number***	***Functional description***
**Clean**	solyc05g047590.2.1	Pectinesterase
	solyc02g081990.2.1	Pectinesterase
	solyc09g075330.2.1	Pectinesterase
	solyc02g075620.2.1	Pectinesterase
	solyc04g080530.2.1	Pectinesterase family protein
	solyc09g075350.2.1	Pectinesterase
	solyc01g090130.1.1	Pectinesterase
**2 DAI**	solyc12g008530.1.1	Pectinesterase
	solyc04g080530.2.1	Pectinesterase family protein
	solyc03g083870.2.1	Pectinesterase
	solyc05g052540.1.1	Os03g0291800 protein (Fragment)
	solyc01g079180.2.1	Pectinesterase
**5 DAI**	solyc01g109740.2.1	Pectinesterase family protein
	solyc01g099950.1.1	Pectinesterase
	solyc02g080200.2.1	Pectinesterase
	solyc02g014300.1.1	Pectinesterase
	solyc06g034360.1.1	Pectinesterase
	solyc03g083840.2.1	Pectinesterase
	solyc07g043240.2.1	Pectinesterase
	solyc03g083870.2.1	Pectinesterase
	solyc06g009190.2.1	Pectinesterase
**15 DAI**	solyc01g091050.2.1	Pectinesterase
	solyc03g078100.2.1	Pectinesterase family protein
	solyc01g098940.2.1	Pectinesterase
	solyc08g078640.1.1	Pectinesterase
	solyc06g034360.1.1	Pectinesterase
	solyc03g083840.2.1	Pectinesterase
	solyc03g083360.2.1	Pectinesterase
	solyc06g009190.2.1	Pectinesterase

Expansin		
Sample	***Number***	***Functional description***
**clean**	solyc06g051800.2.1	Expansin
	solyc03g115320.1.1	Expansin
	solyc01g090810.2.1	Expansin protein
	solyc02g088100.2.1	Expansin
	solyc10g086520.1.1	Expansin-1
	solyc06g005560.2.1	Expansin-1
	solyc04g081870.2.1	Expansin
	solyc09g010860.2.1	Expansin
**2d**	solyc01g090810.2.1	Expansin protein
	solyc08g077900.2.1	Expansin-like protein
	solyc10g086520.1.1	Expansin-1
	solyc03g093390.2.1	Expansin protein
	solyc10g084780.1.1	Expansin
	solyc04g081870.2.1	Expansin
	solyc12g089380.1.1	Expansin
	solyc09g010860.2.1	Expansin
**5d**	solyc03g115320.1.1	Expansin
	solyc08g077900.2.1	Expansin-like protein
	solyc08g007780.2.1	Expansin-like protein
	solyc03g115310.1.1	Expansin
	solyc10g008440.2.1	Expansin B1
	solyc10g086520.1.1	Expansin-1
	solyc03g093390.2.1	Expansin protein
	solyc08g080060.2.1	Expansin-like protein
	solyc09g010860.2.1	Expansin
**15d**	solyc01g090810.2.1	Expansin protein
	solyc08g077900.2.1	Expansin-like protein
	solyc02g088100.2.1	Expansin
	solyc10g086520.1.1	Expansin-1
	solyc03g093390.2.1	Expansin protein
	solyc04g081870.2.1	Expansin

Cellulose synthesis		
Sample	***Number***	***Functional description***
**clean**	solyc03g114900.2.1	COBRA-like protein
**2d**	solyc09g009010.2.1	Cellulose synthase-like C1-2 glycosyltransferase family 2 protein
	solyc02g072240.2.1	Cellulose synthase
	solyc07g005840.2.1	Cellulose synthase 3
	solyc09g072820.2.1	Cellulose synthase
	solyc03g070440.2.1	COBRA-like protein
	solyc03g114900.2.1	COBRA-like protein
	solyc09g075540.1.1	COBRA-like protein
**5d**	solyc08g005280.1.1	Cellulose synthase-like protein
	solyc02g072240.2.1	Cellulose synthase
	solyc09g072820.2.1	Cellulose synthase
	solyc03g114900.2.1	COBRA-like protein
	solyc01g103860.2.1	Ch-cobra
	solyc02g080250.1.1	COBRA-like protein
**15d**	solyc09g009010.2.1	Cellulose synthase-like C1-2 glycosyltransferase family 2 protein
	solyc07g043390.2.1	Cellulose synthase family protein expressed
	solyc03g114900.2.1	COBRA-like protein

Pectate lyase and polygalacturonases		
Sample	***Number***	***Functional description***
**clean**	solyc03g111690.2.1	Pectate lyase
	solyc05g005040.2.1	Polygalacturonase
	solyc12g096750.1.1	Polygalacturonase 4
	solyc05g051350.1.1	Rhamnogalacturonate lyase
	solyc09g091430.2.1	Pectate lyase 1-27
	solyc05g005170.2.1	Polygalacturonase 2
	solyc06g068040.2.1	Polygalacturonase
	solyc12g019230.1.1	Polygalacturonase 1
**2d**	solyc05g005040.2.1	Polygalacturonase
	solyc12g019120.1.1	Polygalacturonase
	solyc12g096750.1.1	Polygalacturonase 4
	solyc04g025440.2.1	Polygalacturonase
	solyc05g014000.2.1	Pectate lyase
	solyc03g114240.2.1	BURP domain-containing protein (Fragment)
	solyc12g019130.1.1	Polygalacturonase
	solyc06g068040.2.1	Polygalacturonase
	solyc12g019230.1.1	Polygalacturonase 1
	solyc08g060970.2.1	Polygalacturonase
	solyc08g068150.2.1	BURP domain-containing protein
	solyc12g019140.1.1	Polygalacturonase
	solyc02g062300.2.1	BURP domain-containing protein
**5d**	solyc12g019120.1.1	Polygalacturonase
	solyc12g096750.1.1	Polygalacturonase 4
	solyc02g080910.2.1	Pectate lyase family protein
	solyc12g019180.1.1	Polygalacturonase 7
	solyc03g007940.2.1	Glycoside hydrolase family 28 protein/polygalacturonase family protein
	solyc06g083580.2.1	Pectate lyase 1-27
	solyc09g061890.2.1	Pectate lyase 1-27
	solyc05g014000.2.1	Pectate lyase
	solyc05g005540.2.1	BURP domain-containing protein (Fragment)
	solyc08g014560.1.1	Polygalacturonase
	solyc11g011310.1.1	Rhamnogalacturonate lyase
	solyc08g068150.2.1	BURP domain-containing protein
**15d**	solyc11g011300.1.1	Rhamnogalacturonate lyase
	solyc12g096750.1.1	Polygalacturonase 4
	solyc12g019180.1.1	Polygalacturonase 7
	solyc01g100980.2.1	Polygalacturonase
	solyc06g083580.2.1	Pectate lyase 1-27
	solyc08g068130.1.1	BURP domain-containing protein (Fragment)
	solyc12g019230.1.1	Polygalacturonase 1
	solyc08g068150.2.1	BURP domain-containing protein
	solyc12g019140.1.1	Polygalacturonase
	solyc01g109500.2.1	BURP domain-containing protein
	solyc01g094970.2.1	Glycoside hydrolase family 28 protein/polygalacturonase family protein
