# Supplementary material for: Meloidogyne javanica fatty acid- and retinol-binding protein (Mj-FAR-1) regulates expression of lipid-, cell wall-, stress- and phenylpropanoid-related genes during nematode infection of tomato
Source: BMC Genomics. 2015 Apr 8;16(1):272. doi: 10.1186/s12864-015-1426-3 (PMC4450471; doi:10.1186/s12864-015-1426-3)
Supplement: Additional file 3: Table A3. — Validation of RNA-Seq results by qRT-PCR. [file 12864_2015_1426_MOESM3_ESM.pdf]

Table A3. Validation of RNA-Seq with qRT-PCR. Values given as log2 of the fold change.

ID	***Annotation***	***OE1-KAN1***		***OE2-KAN2***		***OE3-KAN3***		***OE4-KAN4***		
		RNAseq	***qRT-PCR***	***RNAseq***	***qRT-PCR***	***RNAseq***	***qRT-PCR***	***RNAseq***	***qRT-PCR***	
Solyc05g053380.2.1	WRKY transcription factor 31	-2.30	-0.42	-1.99	-1.65	1.60	0.34	-2.02	-2.24	Genes shared by all treatments
Solyc06g053510.2.1	Spermidine synthase	-5.21	-3.58	-2.87	-2.05	-1.88	-0.70	1.21	2.17	
Solyc08g008310.2.1	Long-chain-fatty-acid-CoA ligase	-3.21	-3.08	-2.93	-2.63	-3.52	-2.72	-3.27	-3.23	
Solyc12g096750.1.1	Polygalacturonase 4	-2.32	-2.22	-3.37	-2.12	-1.90	-1.31	-7.01	-4.88	
Solyc09g007020.1.1	Pathogenesis-related protein	1.70	1.49	1.24	0.80	1.07	1.21	1.14	1.03	
Solyc07g009510.1.1	Chitinase	2.56	3.11	2.44	2.16	1.76	3.07	1.05	0.38	
Solyc02g092820.2.1	Indole-3-acetic acid-amido synthetase GH3.8	-2.99	-2.21	-1.80	-1.59	-4.29	-3.38	-5.59	-5.43	
Solyc12g005310.1.1	Auxin-responsive GH3-like	-2.75	-2.27	-1.18	-1.23	-3.60	-2.94	-4.05	-4.11	
Solyc05g055540.1.1	Nodulin family protein	2.94	1.28	1.64	0.79	2.54	1.93	1.99	2.31	
Solyc10g086520.1.1	Expansin-1	-1.23	-1.05	-1.49	-2.20	-1.81	-0.49	-1.02	-0.75	
Solyc05g018770.1.1	Esterase/lipase/thioesterase	-2.58	-0.16	-12.08	-0.29	-10.93	1.49	-4.19	1.41	
Solyc05g053550.2.1	Chalcone synthase			2.88	0.15	1.67	2.21	-1.64	-1.58	Infected treatments only
Solyc06g005210.1.1	Cytochrome P450 like_TBP			2.12	1.53	1.10	0.80	1.17	0.65	
Solyc09g097770.2.1	Cell wall protein			1.24	0.13	1.59	1.84	1.01	0.52	
Solyc08g021820.2.1	Auxin responsive protein			-10.04	-1.08	-2.65	2.10	-4.16	1.13	
Solyc12g049140.1.1	Extensin-like protein Ext1			-2.16	-1.02	-3.51	-2.12	-2.25	-0.38	
Solyc10g008700.1.1	MYB transcription factor			-1.65	-1.42	2.34	3.05	1.86	1.99	
Solyc01g106640.2.1	Pathogenesis-related protein 1			-1.43	-1.35	-2.26	-1.73	-3.63	-1.67	
Solyc06g005310.2.1	MYB transcription factor			-1.30	-1.29	1.64	2.38	1.19	0.86	
Solyc03g005320.2.1	Fatty acid elongase 3-ketoacyl-CoA synthase			-1.22	-2.07	1.12	-2.41	1.41	-3.30	
Solyc07g006710.1.1	Pathogenesis-related protein PR-1			-1.12	-0.09	-2.26	-0.99	-1.16	-0.14	
