# Supplementary material for: Meloidogyne javanica fatty acid- and retinol-binding protein (Mj-FAR-1) regulates expression of lipid-, cell wall-, stress- and phenylpropanoid-related genes during nematode infection of tomato
Source: BMC Genomics. 2015 Apr 8;16(1):272. doi: 10.1186/s12864-015-1426-3 (PMC4450471; doi:10.1186/s12864-015-1426-3)
Supplement: Additional file 4: Table A4. — Forward and reverse primer sequences used for qRT-PCR confirmation of expression levels of differentially expressed genes from the transcriptomic data, and primers sets for internal control genes used to normalize gene expression in the qRT-PCR analysis. [file 12864_2015_1426_MOESM4_ESM.pdf]

Table A4. Forward and reverse primers sequences used for confirmation of values of differential expressed genes from transcriptomic data, through qRT-PCR, along with primers sets for internal control gene used to normalize gene expression throughout the qRT-PCR expression analysis.

Gene	*Number*	*Forward 5'-3'*	*Reverse 5'-3'*	*Pathway involved*	*Source*
Tubulin	NM_001247878.1	ACCATTTGATCTCTGCAACCATG	TTCACAGCCAATTTCCTCAGG	Reference gene	Iberkleid et al. 2013
Actin	U60482.1	ATGTATGTTGCCATCCAGGCT	TGTGGCTGACACGATCTCCA	Reference gene	Iberkleid et al. 2013
18s	BH01957 (NCBI)	GAAACGGCTACCACATCCAAG	CCCCGTGTTAGGATTGGGT	Reference gene	Iberkleid et al. 2013
WRKY transcription factor 31	Solyc05g053380.2.1	CCAAGGAGCTACTATCGTTGCA	GGGCATGGATGTGTGTGAAC	RNA regulation of transcription	This study
Spermidine synthase	Solyc06g053510.2.1	CCACTTGGTTCCATTCCATCC	GCGAGAGACCTCAAACAACGT	Polyamine metabolism	This study
Long-chain-fatty-acid-CoA ligase	Solyc08g008310.2.1	TCTCTCATATCTCCCCTTGGCA	AATCCTATTGAGGCGCCATG	Lipid metabolism	This study
Polygalacturonase 4	Solyc12g096750.1.1	CTATTGGCCCTGGAAACTCG	CCCAACCTAAGCTTCCAATGC	Cell Wall	This study
Pathogenesis-related protein	Solyc09g007020.1.1	TAGGTTGTGGTCGGGCTCTT	TTGTCCGACCCAATTGCC	Biotic stress	This study
Chitinase	Solyc07g009510.1.1	AAGCGTAAGTGTCCCAACAAGC	GCAGCCAGATCCACAGTAGTCAC	Biotic stress	This study
Indole-3-acetic acid-amido synthetase GH3.8	Solyc02g092820.2.1	CCCTCAGTTCAAATTCGTCAGG	GTGTGTAATTCGGACTCATCGG	Hormone metabolism	This study
Auxin-responsive GH3-like	Solyc12g005310.1.1	CGGGAGGCCTATTAGCTCGT	GTTTGTGTAAGGGTCAGGACGTCT	Hormone metabolism	This study
Nodulin family protein	Solyc05g055540.1.1	GGTAGAGTTGTGTCTGGCTTCTTG	TGTTATCGTGAGCATGAGAGGC	Development	This study
Expansin-1	Solyc10g086520.1.1	GCAACCCTGGAAACCCTTC	CTCCATTATCGTTAGGCAAGGC	Cell Wall	This study
Esterase/lipase/thioesterase	Solyc05g018770.1.1	CAGTCATCAATCTGTCTGGCTGTT	TTGATTGTTGCCTCGAAGTCC	Not assigned	This study
Chalcone synthase	Solyc05g053550.2.1	CAACAAGGTTGCTTTGCTGG	AACAACAAGAACTCGAGCACCC	Scondary metabolism	This study
Cytochrome P450 like_TBP	Solyc06g005210.1.1	CCCGCTTCCGATTCATAGAA	GTGTATAAGTGGGAGTCGAAAGGC	Not assigned	This study
Cytochrome P450	Solyc07g052370.2.1	TTGTATCCTCCTCACACATGGC	CCCATCATGGTTTCTGGCC	Miscelaneous	This study
Cell wall protein	Solyc09g097770.2.1	GCTGAGACTACCAATGCGGTG	CTTACGTTTGCCATAGCCCTTG	Not assigned	This study
Auxin responsive protein	Solyc08g021820.2.1	CCCATCAATGCAATCACGAG	GGCCTCTTCAAAACATCGCT	RNA regulation of transcription	This study
Extensin-like protein Ext1	Solyc12g049140.1.1	GGGAATCCAAGTGAAGAAGCAA	GCCATAGTGCATGTAGCAATCAAG	Not assigned	This study
MYB transcription factor	Solyc10g008700.1.1	AAGGACCTGAACTCTTGCTCCA	TCCGAAACTTGGTGGTGCA	RNA regulation of transcription	This study
Pathogenesis-related protein 1	Solyc01g106640.2.1	ACTCTAATGGTCCTTACGGCGA	CCCACAAGTTAACAGCAGCAGT	Biotic stress	This study
MYB transcription factor	Solyc06g005310.2.1	CATCCTGGTCTCAAACGTGGA	TCTATTTCCCCATTTGGAGTGG	RNA regulation of transcription	This study
Fatty acid elongase 3-ketoacyl-CoA synthase	Solyc03g005320.2.1	GCATATTTAGGATGGGAGGTGC	GGATGAGTTGGTACTTGGAACGTC	Lipid metabolism	This study
Pathogenesis-related protein PR-1	Solyc07g006710.1.1	CCATCAATGCCCTCACTCAAC	GAGCTGCATTTTGAGGAGCC	Biotic stress	This study
