# Supplementary material for: Meloidogyne javanica fatty acid- and retinol-binding protein (Mj-FAR-1) regulates expression of lipid-, cell wall-, stress- and phenylpropanoid-related genes during nematode infection of tomato
Source: BMC Genomics. 2015 Apr 8;16(1):272. doi: 10.1186/s12864-015-1426-3 (PMC4450471; doi:10.1186/s12864-015-1426-3)
Supplement: Additional file 5: Table A5. — Primer pairs used for amplification of promoter fragments. [file 12864_2015_1426_MOESM5_ESM.pdf]

Table A5. Overview of primer pair used for promoter sequence amplification

Name of gene (promoter)	*Solyc number*	*Forward (5'→3')*	*Reverse (5'→3')*	*Source*
Lipoxygenase D	Solyc03g122340.2.1	CCCATTCTCTGAGCTCTCTCGCCTTTC	CCAGTCAGCTCCCGGGAATAATGTTCTCTCTTTAAT	This study
Cell Wall Protein	Solyc09g097770.2.1	CGTGCTCTGCAGAGCTCATTCAAGAG	CTGACCTACCCGGGTCTTAATTATTAT	This study
